# Building capacity for genomics in primary care: a scoping review of practitioner attitudes, education needs, and enablers

**DOI:** 10.3389/fmed.2025.1577958

**Published:** 2025-04-30

**Authors:** Kate L. A. Dunlop, Nehal Singh, Amelia K. Smit, April L. Morrow, Julia Steinberg, Anne E. Cust, Meredith Makeham, Carissa Bonner, Bronwyn Terrill, Lynn V. Monrouxe, David Wilkinson, Shailendra Sawleshwarkar, Alan S. Ma

**Affiliations:** ^1^The Daffodil Centre, University of Sydney, a joint venture with Cancer Council NSW, Sydney, NSW, Australia; ^2^Melanoma Institute Australia, The University of Sydney, Sydney, NSW, Australia; ^3^The Centre for Genetics Education, Health Education and Training Institute (HETI) NSW Health, Sydney, NSW, Australia; ^4^Community and Primary Health Care, Faculty of Medicine, The University of Sydney, Sydney, NSW, Australia; ^5^Leeder Centre for Health Policy, Economics and Data, Faculty of Medicine and Health, The University of Sydney, Sydney, NSW, Australia; ^6^Australian Genomics, Melbourne, VIC, Australia; ^7^School of Clinical Medicine, Faculty of Health and Medicine, UNSW, Sydney, NSW, Australia; ^8^School of Health Sciences, The Faculty of Medicine and Health, The University of Sydney, Sydney, NSW, Australia; ^9^The Royal Australian College of General Practitioners Ltd (RACGP), East Melbourne, VIC, Australia; ^10^Academic Education, Sydney Medical School, The University of Sydney, Sydney, NSW, Australia; ^11^Specialty of Genomic Medicine, Faculty of Medicine and Health, The University of Sydney, Sydney, NSW, Australia; ^12^Department of Clinical Genetics, Sydney Children’s Hospital Network, Sydney, NSW, Australia

**Keywords:** genomics, genomics education, education strategies, primary care, general practitioner

## Abstract

**Introduction:**

Improving clinical capacity for genomics in primary care promises to lead to better health, but genomics uptake in the sector is slow and patchy. This review aimed to identify the attitudes of primary care practitioners (PCPs) and the education needs and enablers in applying genomics to inform priorities in education and implementation.

**Methods:**

Searches were conducted across Medline, Scopus, CINAHL, Embase, and Cochrane CENTRAL until November 2023. Barriers and enablers were mapped to the Theoretical Domains Framework and the Genomic Medicine Integrative Research Framework.

**Results:**

A total of 52 studies were included, and the most frequently mapped domains from the Theoretical Domains Framework were ‘Knowledge’ (65.4% of papers), ‘Environmental context and resources’ (40.4%), ‘Skills’ (38.5%), and ‘Social/professional role and identity’ (32.7%). Four key implications were identified: knowledge as a major barrier and enabler, education to build capacity, uncertainty about the role of PCPs, and additional needs beyond education alone.

**Discussion:**

While PCPs are optimistic about genomics, long-standing barriers to delivery in primary care remain. Multifaceted, evidence-based education strategies, including interactive components to change behaviour, will help to address barriers. Clarifying the role of PCPs, referral pathways, and collaboration with tertiary genetics services will further build capacity for genomics delivery in primary care.

## 1 Introduction

Primary care practitioners (PCPs) are increasingly at the forefront of genomics and are in a unique position to enable the widespread application of precision medicine in the community. Recent rapid advances in genomics have led to cheaper and faster genomic testing and screening, and the emergence of new treatments (1), including targeted therapies for cancer, gene therapies, and tailored medication prescribing guided by pharmacogenomics. Clinical trials are also underway for the use of polygenic scores to provide risk-tailored prevention or early detection of common conditions such as heart disease and cancer, as well as for population-based screening for genetic conditions, with the potential to reduce unnecessary interventions and improve healthcare at scale (1, 2).

Improving clinical capacity for genomics in primary healthcare promises to lead to better health, through earlier diagnosis, more targeted risk management, and early intervention (3). Primary healthcare supports first-contact, person-focused care and serves as a strategic entry point to the health system (4). This includes family physicians, general practitioners, nurse practitioners, and physician assistants.

Despite considerable development of genomics education resources for health professionals in the last decade, there has been a relatively slow uptake of genomics into primary care, with many practitioners reporting inadequate capacity, capabilities, training, and support to enable genomics to be embedded into their practice (5, 6). In addition, the rapid pace of genomic advancements has the potential to outstrip updates provided by existing education resources, presenting additional challenges in engaging PCPs in genomics education. Internationally, strategies to support primary care professionals in the delivery of genomics medicine have been proposed (7, 8), but there remains a lack of evidence on the most effective education approaches and key priorities in genomics education and implementation in this sector.

We conducted a scoping review to present a cohesive overview of the attitudes of PCPs to genomics and education needs and enablers in applying genomics in primary healthcare to better understand how to build capacity through education and inform implementation. We defined ‘enablers’ as any factors facilitating the successful implementation of education, such as tailored resources addressing stakeholder needs. Moreover, we have defined primary care practitioners as those that align with the WHO definition of primary care, providing first contact, accessible, continuous, comprehensive, coordinated care that is person-focussed (4). Specifically, as this study is funded through an Australian Medical Research Futures Fund project aimed at finding genomic solutions for general practitioners, we tried to align the definition of primary care practitioner as closely to the Australian system as possible in our search strategy.

This scoping review had two key objectives:

1. To understand the attitudes of PCPs, in particular GPs, toward genomic practice in the context of genomics education and how these can be addressed; and.
2. To examine the evidence on genomics education in primary care to identify what works and the needs of PCPs.

## 2 Materials and methods

The review has been reported in accordance with the Preferred Reporting Items for Systematic Reviews and Meta-Analyses Extension for Scoping Review (PRISMA-ScR) guidelines (9).

### 2.1 Criteria

A detailed search strategy and eligibility criteria for screening of studies were developed in collaboration with the authors and a clinical librarian at the University of Sydney. Studies were included if they met the following criteria:

- Included genomics education and/or resources (excluding out-of-scope topics, i.e., non-genetic newborn bloodspot screening and tumour testing in tertiary setting)
- Reported genomics education needs, gaps, and enablers
- Based on primary care settings involving primary care professionals (e.g., general practitioners, primary care nurses, or equivalent roles in primary care settings outside Australia, such as family physicians and physician assistants)
- Published between 2011 and 2023 and
- Full text was available in English.

Studies were excluded if primary care professional roles and responses were not clearly differentiated from professionals in other health sectors, such as tertiary care, in the data analysis. For example, if a study interviewed PCPs and surgeons and included all their responses mixed without differentiation, these were excluded. This was to ensure that we only had responses purely from primary care professionals, so that relevant barriers and enablers could be attributed to evidence from primary care practitioners. This means we deliberately excluded studies performed in the tertiary care sector due to the different health system issues in this field, and also there is a relative abundance of studies looking at mainstreaming in tertiary care (10). We excluded protocols, conference abstracts, commentaries, letters, editorials, or perspectives and studies not available in English.

### 2.2 Information sources and search

Searches were conducted across five databases, Medline, Scopus, CINAHL, Embase, and Cochrane CENTRAL, to capture all relevant literature published on the research topic from the genomic era, January 2011 until November 2023. Search strategies and terms used across the different databases are available in Supplementary Table 1.

### 2.3 Selection of sources of evidence

All studies were analysed for relevance to the objectives using the eligibility criteria. All studies were initially uploaded to Endnote, a citation and reference management tool, where duplicates were removed from the library. Covidence, a systematic review management software, was used to screen studies to be included in the scoping review. The studies captured via the database searches were uploaded to Covidence to commence screening studies based on the relevancy of the abstract and title. This stage was completed by two authors (NS and KD), with a subset of articles (10%) reviewed by both to ensure screening reliability before commencing the full set of studies. A second screening was automatically completed at this stage by Covidence to verify that all duplicate studies were excluded before the authors (NS and KD) independently commenced screening the select studies based on their full texts. Consensus on the inclusion of studies was met through weekly discussions throughout the screening process; any conflicts were resolved by discussions with senior authors in the team (ASM and AS) until fully agreed.

### 2.4 Data charting process and items

Data were extracted, and studies were charted in a table format by two authors (NS and KD), documenting article details (title, author, year of publication, and country of publication), study summary (aims, methods, and results), study details (participants, specialty within genomics, type of intervention, and key outcome measures), and outcomes (needs, gaps, barriers, and facilitators). Studies were categorised according to their key focus, either attitudes of PCPs toward practicing genomics or educational interventions (genomics).

### 2.5 Synthesis of results

The selection, screening, and synthesis of studies in this review was completed in 12 months. Studies were further coded to two frameworks and discussed in regular meetings over the following 6 months to provide a structured approach for deductive analysis (barriers and enablers) and inductive analysis to determine implications as follows:

- Behavioural Domains using the Theoretical Domains Framework (TDF): The TDF has been applied across a broad range of healthcare settings and behaviours to categorize barriers and enablers, including in genomics uptake (11). This framework provides a comprehensive structure to understand determinants of behaviour change relevant to the education delivery of genomics in primary care. Each of the 14 domains of TDF was defined by the study team, with examples in the context of this review available in Supplementary Table S3. These domains were mapped initially by two authors (NS and KD) and further correlated by a third author to ensure consistency (AM). Barriers and enablers were further coded for each article according to attitudes-based or educational intervention-focused studies in line with the aims of the review. Any discrepancies were discussed among five co-authors (NS, KD, ASM, AKS, and ALM) at regular research meetings. A frequency analysis of domains was completed by one author (NS) to further guide understanding of the key genomics-related gaps and needs of PCPs prevalent in the literature (included in Supplementary Table 3 with TDF domain definitions).
- The Genomic Medicine Integrative Research Framework (GMIR): GMIR (12) is a conceptual framework to help design measures for integrating genomics into clinical practice. TDF barriers, enablers, and needs were coded into the GMIR to capture contextual factors, educational interventions, processes, and outcomes to guide further analysis (12) (included in Supplementary Tables 4, 5). Findings within the GMIR were checked and discussed amongst authors, including the impact of genomics education approaches and how they play out in the real world of primary care. A discussion was conducted at regular research meetings (NS, KD, ASM, AKS, and ALM) and at two additional meetings with all authors whose range of academic backgrounds include epidemiology, clinical genetics, implementation science, genomics education, health professional education, and primary care. This enabled the determination of implications for building capacity for genomics in this setting. This scoping review provided a high-level map of existing literature and knowledge, and so a critical appraisal of individual studies was not conducted.

## 3 Results

**Figure 1:**
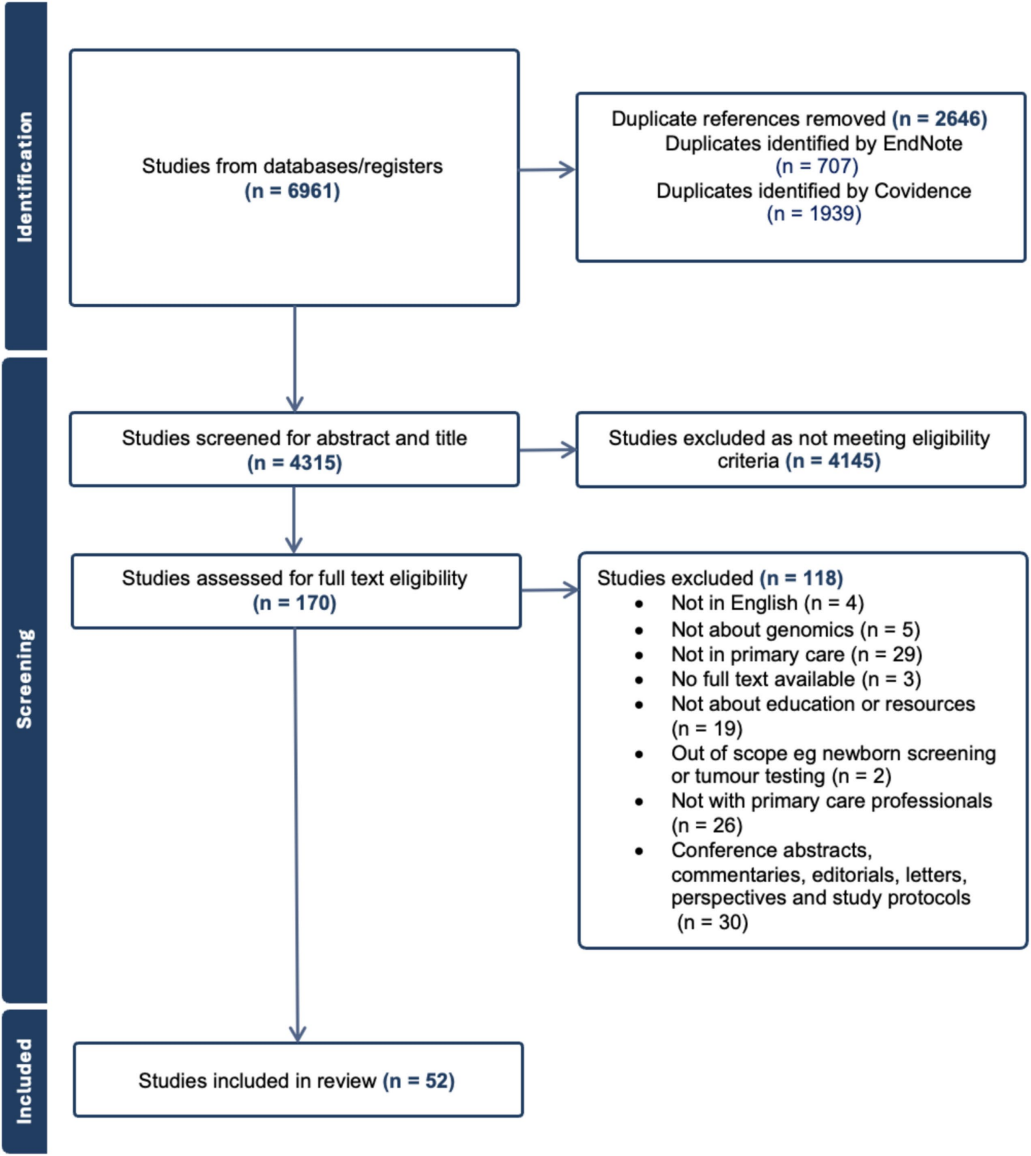
PRISMA diagram illustrating article screening process.

After all duplicates were removed, a total of 4,315 studies were screened for abstract and title (Figure 1). After excluding studies that did not meet the eligibility criteria, a total of 170 papers were assessed in full text, and 52 were included in the review, with 33 focused on attitudes of PCPs and 19 on educational interventions (genomics).

Demographic information and study characteristics of all included studies are shown in Supplementary Table 2. All included studies with other extracted data items are shown in Table 1 (those with a focus on attitudes and views of primary care practitioners) and Table 2 (those with a focus on educational interventions for primary care practitioners).

**Table 1:** All included studies with a focus on attitudes and views of primary care practitioners.

Author (Year)	Country	Study aim	Methods	Genomics topic	Key outcomes
Cusack (2021) (5)	Australia	To identify Australian general practitioners’ views on genomics, impact on practice and education needs to inform continuing education.	Interviews	Prenatal screening and single gene tests.	Views on genomics, practice, and continuing education.
Mitchell (2022) (24)	Switzerland	To investigate the current level of knowledge of precision medicine, acceptable content for training, the perceived potential of a more precision approach to patient care, and motivation to participate in a training programme.	Focus groups	Precision medicine	Acceptability of a training programme.
Best (2023) (20)	Australia	This study aimed to identify and prioritise implementation strategies to reduce barriers and support healthcare practitioners to routinely offer reproductive genetic carrier screening in Australia.	Survey	Reproductive genetic carrier screening	Barriers before offering reproductive genetic carrier screening, supports that could facilitate healthcare practitioners offering screening, and prioritised supports toward the end of the study analysed specialty and clinic locations separately.
Best (2023) (30)	Australia	To identify influences on healthcare professionals considered as ‘early adopters’ offering reproductive genetic carrier screening through Mackenzie’s missions, an Australian national research study investigating the implementation of free reproductive genetic carrier screening to couple’s preconception or in early pregnancy.	Interviews	Reproductive genetic carrier screening	Healthcare practitioners perceived barriers and enablers to offering reproductive genetic carrier screening.
Bernhardt (2012) (15)	USA	To assess primary care practitioners current experience with genetic testing, their assessment of the understandability and clinical utility of information in sample direct-to-consumer reports for genomic assessment of disease risk and warfarin dosing and attitudes toward genomic medicine.	Survey	Direct-to-consumer genomic testing	Responses to reports of direct-to-consumer genomic testing and attitudes toward personalised genomics.
Carroll (2019) (14)	Canada	To determine family physicians’ current involvement and confidence in genomic medicine, attitudes regarding its clinical value, suggestions for integrating genomic medicine into practice, and resources and education required.	Survey	Genomic medicine	Family physicians’ current involvement with genomic medicine in practice.
Carroll (2016) (50)	Canada	To assess primary care practitioners’ experiences with perceptions of and desired role in personalised medicine, with a focus on cancer.	Focus groups	Personalised medicine	Experiences with personalised medicine.
Carroll (2021) (51)	Canada	To explore genetic health professionals’ expectations of primary care professional’s role in genomic medicine now and in the future.	Focus groups	Genomic medicine	Practitioner expectations with genomic medicine.
Evans (2020) (54)	UK	To capture which education approaches are currently used for genomic clinical scenarios and to develop a greater understanding of the resources utilised for certain specific resources (resources to support clinicians looking after rare disease patients, direct-to-consumer genetic testing, and collecting family history).	Survey	General genomic information	Utility of current resources.
Fok (2021) (23)	Singapore	To explore family physicians’ attitudes, perceived roles, motivators, and barriers towards genetic screening and to explore similarities and differences between private and public sector family physicians.	Interviews	Genetic screening	Family physicians’ attitudes, perceived roles, motivators and barriers towards genetic screening.
Haga (2012) (78)	USA	To seek primary care practitioners’ views on their willingness and readiness to utilise pharmacogenetic testing, desirable test properties, and factors relevant to the use of pharmacogenetic testing.	Surveys	Pharmacogenetic testing	Primary care practitioners training, familiarity and attitudes toward pharmacogenetic testing.
Harding (2019) (21)	Canada	To explore the self-identified needs, including education needs, of both urban and rural primary care practitioners to provide genetic care to their patients.	Mixed methods	Overall genetic care	Self-identified genetic needs of primary care practitioners with specific consideration paid to the unique needs of both urban and rural primary care practitioners.
Hauser (2018) (13)	USA	To survey primary care practitioners to assess their attitudes and beliefs, generally about genetic testing and specifically for common chronic diseases.	Survey	Genetic testing for chronic diseases	Insights for the sustainable adoption and large-scale dissemination of genomic medicine, both broadly and for diverse clinical settings and ancestral populations.
Houwink (2012) (33)	Netherlands	To prioritise topics for genetics education for general practice.	Delphi/Workshops	Overall genetics	Priority topics for genetics education in general practice.
Houwink (2011) (34)	Netherlands	To explore the role of genetics in primary care (i.e., family medicine and midwifery care) and the need for education in this area as perceived by primary care practitioners, patient advocacy groups, and clinical genetics professionals.	Focus groups	Overall genetics	Exploration of the meaning and significance of the role of genetics and the need for education in that area as perceived by different stakeholders.
Jamterud (2021) (18)	Netherlands	To present an empirical bioethics analysis of the preconception expanded carrier screening practice from the perspective of general practitioners.	Interviews	Preconception expanded carrier screening	Examined general practitioners’ views and/or experiences on the practice of preconception expanded carrier screening covering: first impression of the test, implications of the test, experiences with patients. and how the test could be improved.
Lemke (2020) (17)	USA	To elicit primary care practitioners’ perceptions of and experiences with incorporating large-scale genetic testing into their clinical practice.	Mixed methods	Genetic testing	Perceived value of and barriers to incorporating genetic testing into the clinical practice of primary care practitioners.
Rafi (2020) (52)	UK	To explore the potential barriers, opportunities, and challenges facing the implementation of pharmacogenetic testing into primary care.	Interviews	Pharmacogenetic testing	Barriers, opportunities, and challenges facing the implementation of pharmacogenetic testing into primary care.
Sebastian (2022) (79)	Canada	To explore primary care providers’ challenges and potential solutions for managing secondary findings from genomic sequencing.	Interviews	Genomic sequencing	Challenges and solutions managing secondary findings from a hypothetical patient or patient in practice.
Smit (2019) (27)	Australia	To explore general practitioners’ attitudes toward communicating genomic risk information and resources needed to support this process.	Interviews	Genomic risk information	Attitude toward communicating genomic risk information and resources needed to support this process.
Vassy (2023) (49)	USA	To understand the perceived clinical utility, benefits, and barriers to using polygenic risk scores in preventive care.	Surveys	Polygenic risk scores	Physicians’ medical decision-making with polygenic risk scores and perceived benefits and barriers to polygenic risk testing.
Wilson (2016) (28)	Canada	To use the theory of planned behaviour as a lens to examine the behaviours underlying cancer genetics referral decision-making by family physicians and to clarify whether tailoring continuing medical education interventions might offer a useful way forward to support the implementation of genetics in primary care.	Surveys	Genetics referral decision-making	Examination of attitude, subjective norms, and perceived behaviour control which all inform intention and behaviour for referrals.
Yu (2021) (19)	Hong Kong and Shenzhen, China	The aim was to evaluate knowledge, attitudes, and clinical practice concerning medical genetics, genetic testing, and counselling among primary care practitioners in Hong Kong and Shenzhen, China.	Surveys	Common genetic diseases	Knowledge (understanding of disease), attitudes (and opinion on usefulness), confidence, and training needs in genetic and related areas.
VanViet (2023) (47)	The Netherlands	To explore strategies for hemoglobinopathies screening in the preconception phase in high-risk patients. Needs for education and communication with patients and their families are explored.	Interviews	Hemoglobinopathies	General practitioners’ knowledge and communication around hemoglobinopathies.
VanWyk (2016) (31)	South Africa	To assess the practices, knowledge, and attitudes of general practitioners regarding common hereditary cancers.	Surveys	Hereditary cancers	Knowledge, management of at-risk patients, and attitude toward learning more about inherited cancers and relevant services.
Ayoub (2023) (26)	UK	This study aimed to explore general practitioners’ knowledge of risk-stratified screening; attitudes toward risk-stratified screening; and preferences for continuing professional development.	Surveys	Polygenic risk scores and risk-stratified population screening	General practitioners’ knowledge, attitudes, and preferences for continuing professional development.
Baroncini (2015) (29)	Italy	To explore knowledge/awareness, involvement, and attitudes of primary healthcare providers on direct-to-consumer marketing of personal genomic tests.	Surveys	Direct-to-consumer personal genomic tests	Awareness and attitudes of general practitioners toward direct-to-consumer personal genomic tests.
Leitsalu (2012) (16)	Estonia	To assess primary care practitioners’ knowledge base in genetics and review their opinions on how to incorporate genomic risk assessment into healthcare.	Surveys	Genetics	Clinical use of genetic information in practice, genomic information, and predictive testing, informing patients of risks, ethical and social ramifications, and opinions of and ideas for training programs.
Marathe (2015) (22)	Australia	To investigate the knowledge and management of genetic cardiac diseases by general practitioners.	Surveys	Genetic cardiac diseases	Management of genetic cardiac diseases in practice, the importance of patient education and practitioner confidence to deliver patient education, opportunities available to general practitioners for genetic counselling, and practitioners perceived importance of multidisciplinary care and support of patients with genetic cardiac diseases.
Melo (2015) (25)	Brazil	To analyse genetic care competencies of primary care practitioners in Brazil.	Surveys	Genetics	Core competencies for genetics in primary care practitioners.
Nair (2017) (32)	USA	To identify knowledge gaps in hereditary breast and ovarian cancer syndrome inheritance patterns and identification of high-risk families.	Surveys	Hereditary breast and ovarian cancer	Hereditary breast and ovarian cancer knowledge and provider confidence regarding knowledge of hereditary breast and ovarian cancer, genetic counselling referral practice patterns, prior participation in continuing medical education activities related to cancer genetics, and interest in additional hereditary breast and ovarian cancer education.
Skinner (2021) (35)	Canada	To compare the performance of Canadian family physicians to Canadian genetic counsellors regarding the interpretation and management of genetic testing results.	Surveys	Genetic testing	Number of correct responses to the genetics knowledge questionnaires.
Tan (2014) (48)	Australia	To assess Australian clinicians’ knowledge, attitudes, and referral patterns of patients with suspected Lynch syndrome for genetic services.	Surveys	Lynch syndrome	Referral practices, barriers, and motivators for genetics referral, physician referral preferences, and perceptions of their role and their desired support for the provision of genetic services.

**Table 2:** All included studies with a focus on educational interventions for primary care practitioners.

Author (Year)	Country	Study aim	Methods	Genomics topic	Key outcomes
Terrill (2024) (36)	Australia	The aim was to evaluate the effectiveness of an e-learning module to increase general practitioner awareness and knowledge of genomics, increase confidence, and foster intention.	Mixed methods	Genomic testing	Learning outcomes met from the course and impact of the modules on behavioural intentions.
Vieira (2013) (37)	Brazil	To ascertain whether implementation of a medical genetics’ education program produced for primary care providers could contribute to the integration of concepts and attitudes related to the identification, management, and prevention of congenital malformations and genetic diseases into the care provided at primary healthcare units.	Surveys	Genetic services	Practitioner scores on survey questions to measure changes by education program.
Telner (2017) (39)	Canada	To evaluate and compare the impact of three methods of delivering primary care genetic content to family medicine residents.	Randomised controlled trial	Genetics	Practitioner scores on knowledge, attitudes, and skills.
Houwink (2014) (40)	The Netherlands	To measure the educational outcomes of an oncogenetics electronic continuous professional development module for satisfaction, knowledge, and knowledge retention.	Randomised controlled trial	Oncogenetics	Satisfaction with the training module, overall knowledge, and knowledge retention.
Houwink (2015) (44)	The Netherlands	To give an overview of a research project on how to build effective educational modules on genetics and to investigate the long-term increase in genetic consultation skills (1-year follow-up) and interest in and satisfaction with a supportive website on genetics among general practitioners.	Mixed methods	Oncogenetics	Self-reported genetic competencies and changes in referral behaviour, referral rates from general practitioners to clinical genetics centres, and satisfaction and website visitor count a year post education.
Houwink (2014) (46)	The Netherlands	To investigate whether oncogenetics training for general practitioners improves their genetic consultation skills (1-month and 3-month post-training).	Randomised controlled trial	Oncogenetics	Satisfaction with the face-to-face training and applicability of the new consultation skills.
Carroll (2011) (38)	Canada	To determine if a multifaceted knowledge translation intervention would improve skills, including referral decisions, confidence in core genetic competencies, and knowledge.	Randomised controlled trial	Genetics	Number of genetic referrals post-intervention.
Dormandy (2012) (80)	UK	To evaluate brief communication skills training for primary healthcare professionals in offering antenatal sickle cell and thalassaemia screening in primary care.	Randomised controlled trial	Sickle cell and thalassaemia screening	Attendance, perceived usefulness of training, comfort and confidence in offering screening, offering screening at pregnancy confirmation consultations, and gestational age at test uptake.
Westwood (2012) (55)	UK	To test whether primary care genetic-led genetics education improves both non-cancer and cancer referral rates and whether primary care-led genetics clinics improve the patient pathway.	Randomised controlled trial	Cancer (breast, colorectal, ovarian) and non-cancers (cystic fibrosis, Huntington’s disease)	Number of genetic referrals post-intervention.
Brown-Johnson (2021) (81)	USA	To assess implementation outcomes, specifically penetration/reach, acceptability, feasibility, and sustainability to inform future implementation initiatives and facilitate scale/spread of precision health in primary care. Early potential clinical benefit was also assessed to patients.	Randomised controlled trial	Precision Medicine	Penetration/reach, acceptability, feasibility, and sustainability of the intervention (along with other implementation outcomes).
Barreiro (2013) (56)	Argentina	To implement a model (CAPABILITY ARGENTINA outreach project) to introduce genetics in areas without genetic services and become part of primary care.	Mixed methods	Genetic healthcare	Recommendations in the implementation of a program, participation in training, and genetic consultation rates.
Bell (2015) (43)	USA	To evaluate the outcomes of an interactive web-based genetics curriculum versus a text curriculum for primary care physicians.	Randomised controlled trial	Genetics	Effectiveness of education intervention on appropriate physician behaviours and covering topics.
Calabro (2021) (42)	Italy	To investigate the effectiveness of a distance learning course on genetics and genomics targeted at medical doctors.	Mixed methods	Genetics and genomics	Effectiveness of a distance learning course on genetics and genomics targeted at medical doctors.
Carroll (2016) (76)	Canada	To determine the value of Gene Messengers as a continuing education strategy in genomic medicine for family physicians.	Surveys	Genetic testing	Cognitive impact, relevance of intervention, and intended use of information for a patient and expected health benefits.
Carroll (2014) (45)	Canada	To determine if the colorectal cancer risk triage/Management tool would enable family physicians to appropriately triage and make screening and genetics referral recommendations for patients with colorectal cancer family history.	Surveys	Colorectal cancer	Mean change in score (sum of “correct” responses) for the following: colorectal cancer risk category, screening method, age to start screening, frequency of screening, and decision to refer to genetics for the eight clinical vignettes. Secondary outcomes included decisional difficulty around colorectal cancer risk assessment, confidence in primary care genetic skills, and responses to the usefulness of the tool.
Hansen (2024) (77)	USA	To evaluate participating primary care practitioners’ perceptions of the program’s education modalities and to assess the program’s impact on primary care practitioners’ confidence in navigating genetic disease screening as part of their clinical practice.	Surveys	Genetic screening	Current engagement and growth in perceived clinical genetics competency, utility of existing program educational resources, and ideas for educational improvements.
Jackson (2014) (82)	The UK/The Netherlands	To develop (i) guidelines for potential consumers who are considering using direct-to-consumer genetic tests and (ii) guidance for health professionals who are approached by patients who are considering or have already undertaken such tests.	Workshops	Direct-to-consumer testing	Clinically relevant and pragmatic guidance for patients and health professionals in the form of a decision support tool for use in primary care.
Jackson (2019) (41)	The UK/The Netherlands	To evaluate a series of e-learning resources to equip primary care professionals with genetic skills relevant for practice using Kirkpatrick’s framework for educational outcomes.	Mixed methods	Genetics	Satisfaction with training in terms of changes in knowledge and skill, in perceived confidence in providing genetic healthcare, in self-reported clinical practice behaviour, and in affecting the wider profession or healthcare community.
Presutti (2023) (53)	USA	To understand the extent to which primary care practitioners use cancer-related family history questionnaires to refer patients for genetic testing.	Surveys	Genetic testing	The main outcome was the percentage of primary care practitioners who identified each question as a trigger for genetic testing. Secondary outcomes included correlations with years of practice, genetics training, and methods used to obtain patient family history.

Attitudes of PCPs were optimistic about the potential for genomics to improve clinical care (13–19) and as an area of responsibility for primary care (5, 20, 21). However, most reported low skill and knowledge (13, 19, 22, 23), in particular in referral pathways and dealing with complexities of genomics (5, 24, 25), lack of confidence, especially in counselling and interpreting genomic results (5, 14, 17, 22, 26–29), and poorly defined roles (24). There was strong interest amongst PCPs in genomics education (21, 30–32).

Barriers, enablers, and needs were most frequently mapped to the TDF domain ‘Knowledge’ in 34/52 (65.4%) of articles, followed by ‘Environmental context and resources’ in 21 papers (40.4%). Barriers and enablers were also frequently categorised into skills (38.5%), social/professional role and identity (32.7%), beliefs about capabilities (17.3%), memory, attention, and decision processes (13.5%), optimism (11.5%), intentions (9.6%), and beliefs about consequences (7.7%). Mapping to other TDF domains occurred on one occasion or not at all. A summary of the barriers, enablers, and needs for the key TDF domains, categorised according to attitudes-focused or educational intervention-focused studies, is included in Table 3. All 14 TDF domains were relevant in this scoping review, and the remaining domains are included in Supplementary Tables 3–5.

**Table 3:** Summary of key barriers, enablers, and needs in primary care for five common TDF domains [according to attitudes to genomics or educational interventions].

TDF domain	Barriers	Enablers	Needs
Knowledge (and Practitioner skills)*	Attitudes to genomics Most primary care practitioners report low skill, knowledge, and experience with genetics (13, 19, 22, 23). This theme is repeated in many subcategories of genetics and includes a lack of knowledge on appropriate referral pathways for genetic patients and dealing with complexities of genomics (5, 14, 24, 25, 30, 76).	Attitudes to genomics There is interest amongst primary care practitioners for more education in this area, including continuous professional development activities (30), engagement with experts (20), and clearer referral pathways in genetics (21). The majority of general practitioners report interest in further education (31, 32).	Continuous professional development and online multifaceted resources are needed, with a diverse range of methods (5). This includes better training curricula (25, 32) covering the basics of genetics, psychosocial issues, and referral indications (33–35). Evidence-based education strategies, i.e., content informed by target group, based on case studies, multiple methods, tools, interactive components, and reflective learning to address skills needs.
	Educational interventions Standalone lectures/resources rarely increase knowledge (37, 38). Web-based interventions alone are unlikely to impact behaviour change (43, 46).	Educational interventions Problem-based, case-based online learning interventions increase knowledge (36, 39–42). Interactive scenarios move knowledge into practice, e.g., especially if included skills/ role play with interactive elements. Sustained improvement in consultation skills 3 months after face-to-face skills and role-play training (46) (TDF: Skills).	
Environmental context and resources	Attitudes to genomics Lack of time reported by many primary care practitioners (19, 20, 30) for the counselling and discussion of genetics required. Moreover, resource issues: lack of financial support (24) and access to genetics advice (51, 52).	Attitudes to genomics Funding for time spent in genetics (30), as well as improved referral guidelines and patient information and links to local genetics services support (20), could address the lack of time/resources (26).	Better awareness of appropriate genetic referral pathways (22, 47, 48, 51, 54), supports, and resources to enable genomics, including patient information required. Education and information resources need to be accessible, brief, and funded for sustainability. Providing a supportive learning workplace/ environment may help put knowledge into practice for the delivery of genomics. General practitioners need live education to improve appropriate referrals and ideally access to a community of practice in genomics (30, 51).
	Educational interventions Expansion/sustainability of education programs is limited by cost constraints and the availability of human resources (37).	Educational interventions Convenience, time, and pace of web-based modules appreciated, e.g., gene messenger (76). Availability of resources for teaching also serves as a vehicle to form stronger links between primary care and genetics (41). Furthermore, having access to a community of practice or multidisciplinary team model (36) or being part of a reproductive genetic carrier screening special interest group(30)was suggested. General practitioners’ who attended live training were more likely to consider referring patients to clinical genetics centres (44) and having resources available, i.e., colorectal cancer risk triage tool, significantly increased confidence in referral (45).	
Professional role and identity	Attitudes to genomics Poorly defined roles in genetics for primary care practitioners (24), including ambivalence (23) and uncertainty in the profession toward genomics (47, 48).	Attitudes to genomics Many primary care practitioners see a growing role for genomics in their practice as an area of responsibility for them (5, 20, 21).	Clearer role delineation is needed to demonstrate how primary care practitioners play a part in the genomics journey, including recognition of general practitioners’ roles in genomics and mainstreaming. General practitioners favoured the inclusion of case studies modelling pivotal roles for general practitioners, such as taking an accurate family history and referring appropriately to genetics.
	Educational interventions	Educational interventions Genetics Health professionals presenting education to primary care enables greater appreciation of roles (37).	
Beliefs about capabilities	Attitudes to genomics Lack of confidence is reported in many primary care practitioners, especially in counselling and interpreting genomic results and advising patients (5, 14, 17, 22, 26–29).	Attitudes to genomics Despite low confidence, many primary care practitioners express optimism that genomics will be useful, improve clinical care, and make a positive impact (13–16).	Distilling information into a useful and accessible “bottom line” with which to guide practice: e.g., ‘Genomics fundamentals’ accessible as a refresher (36) that could be accessed anytime and before undertaking e-modules, including fundamental genomics topics of genomic testing, genetic variation, and genetic inheritance.
	Educational interventions Primary care professionals report low confidence in delivering genomics, particularly communicating genomic information to patients (41).	Educational interventions Evidence-based education interventions increased confidence, e.g., Genetikit evidence-based summaries (38), colorectal cancer Risk Triage tool (45), Genomic Medicine Action Plan messaging tool (77), Gen-equip (36, 41).	
Beliefs about consequences	Attitudes to genomics Primary care practitioners report concerns about negative impact on patients including anxiety, insurance, discrimination, costs, and privacy (13, 16, 17, 20, 24, 49).	Attitudes to genomics Many primary care practitioners see genomics as improving patient care (17), offering better reproductive choices (18), and personalized medicine (19).	Negative concerns about genomics need to be addressed clearly for primary care practitioners and patients, and the potential benefits of genomics.
	Educational interventions Online education may not change attitudes including module and live webinars (39).	Educational interventions General practitioners’ expected health benefits after an interactive intervention with reflective learning (50).	

^*^Domains were combined to allow for overlap.

### 3.1 Implications for building capacity for genomics in primary care

**Figure 2:**
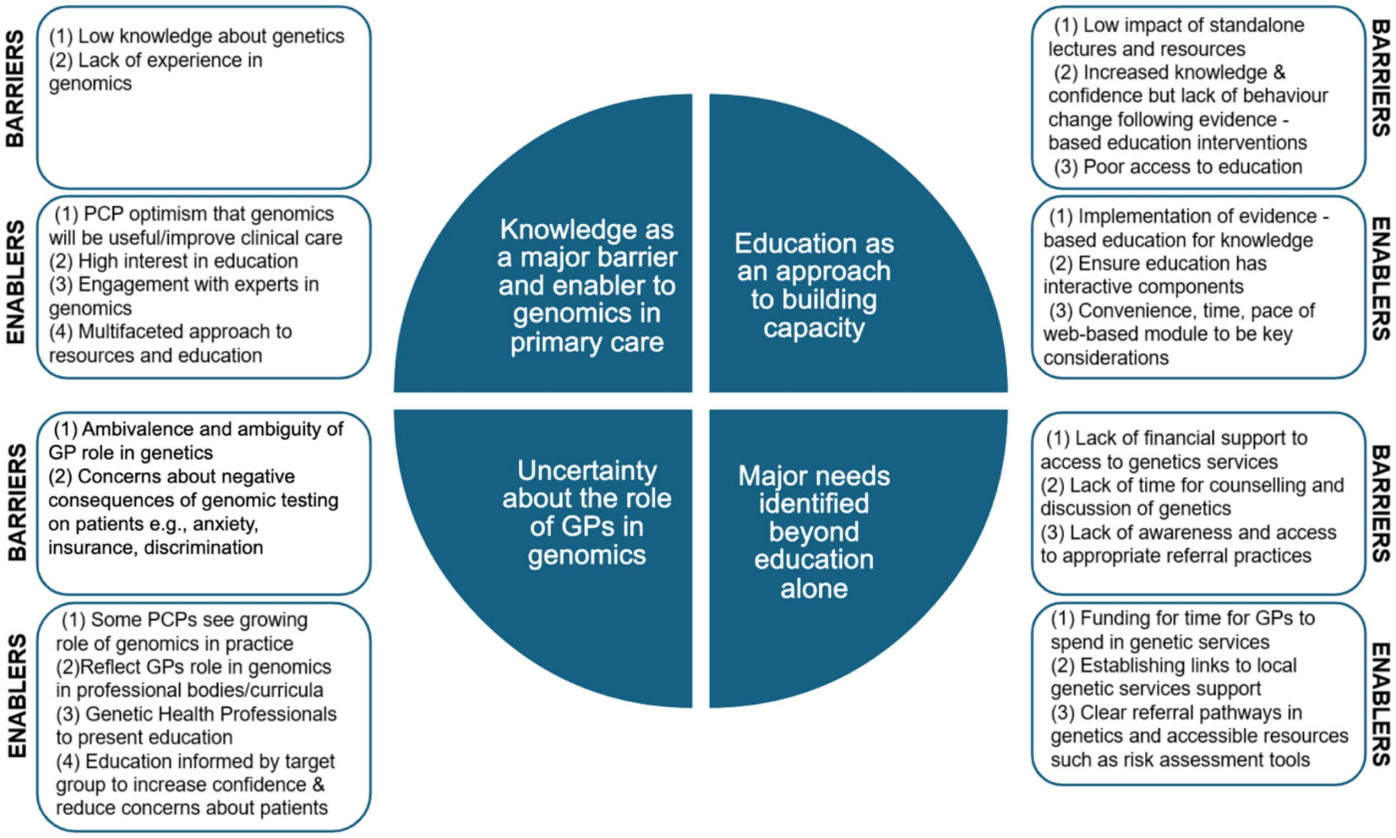
Implications for building capacity for genomics in primary care.

Four key implications were identified from the data analysis of included studies as follows and summarised in Figure 2.

#### 3.1.1 Knowledge as a major barrier and enabler to genomics in primary care

Limited knowledge about genomics and lack of experience in genomics was a frequently reported barrier (TDF: Knowledge) to delivering genomics in primary care, amplified by the complexities of genomics (5, 14, 24, 25, 30). This was linked closely to the barrier of PCP’s low confidence and perceived ability to perform genomics in practice, such as counselling families and explaining genomic results (5, 14, 17, 27–29) (TDF: Beliefs about capabilities). Key enablers included PCP’s optimism that genomics will be useful, improve clinical care, and make a positive impact (13–16) and the high interest in further education (20, 31, 32). Other enablers included engagement with experts (20).

The need for continuous professional development (CPD), accredited and multifaceted in approach (using multiple modalities, e.g., online, face-to-face, workshops, and modules), was identified to meet the range of PCP’s needs and preferences for delivery (5). These include better training curricula (25, 32) covering the basics of genetics, psychosocial issues, referral indications (33–35), and useful ‘bottom-line’ information accessible anytime to help build confidence (36).

#### 3.1.2 Education as an approach to building capacity

There were two overarching barriers to educational interventions for building capacity for genomics. The first included the low impact of standalone lectures and resources, with two articles reporting knowledge was not retained after lecture series (37) and evidence-based summaries (38) (TDF: Knowledge). Implementing education informed by evidence-based strategies to increase confidence and knowledge (TDF: Knowledge) was a key enabler. For example, several studies reported that active problem-based, case-based online learning interventions were effective evidence-based strategies, increasing genomic knowledge for PCPs (36, 39–42).

The second barrier identified was the limited evidence of behaviour change despite PCPs participating in evidence-based educational interventions (40) (TDF: Knowledge). Significant impact on applying knowledge, for example, key counselling behaviours, was not achieved following participation in an e-module (40) and a web-based genetic curriculum (43). Improvement in self-reported genetic competencies and referral behaviour at 1-year follow-up was reported by PCPs who completed comprehensive oncogenetic training (44) (a module, live education, and a website), though clinical genetics centres reported no significant change in referral numbers 1 year after the training.

Despite this, PCPs did frequently report an intention to implement support for genomic testing in practice following such interventions (36, 44, 45), and behaviour change was achieved in a small number of studies that included an interactive education component. Sustained improvement in consultation skills was reported by PCPs 3 months following interactive face-to-face skills and role-play training (46). An e-learning tool that provided evidence-based summaries of new genetic tests with primary care recommendations, while not improving knowledge, increased confidence and changed practice with participants choosing to continue to receive the resource (46). Poor access was reported by some as a deterrent to participating in education, with convenience, time, and pace of web-based modules as recognised enablers (39).

#### 3.1.3 Uncertainty about the role of GPs in genomics

Poorly defined roles in genetics for PCPs (24) were seen as barriers to the delivery of genomics, including ambivalence (23) and uncertainty in the profession toward genomics (47, 48) (TDF: Professional Role and Identity). Enablers included that some PCPs see a growing role for genomics in their practice as an area of responsibility for them (5, 20, 21). Strategies to clarify the role of the GP in genomics, such as reflecting the PCP’s role in activities provided by professional bodies and training curricula to provide baseline knowledge (36), were also reported. Genetic health professionals presenting education to primary care may enable a greater appreciation of roles (37).

Concerns about the negative consequences of genomic testing on patients, including anxiety, insurance, discrimination, costs, and privacy (13, 16, 17, 20, 24, 49), were also key barriers (TDF: Beliefs about consequences). A key enabler to increase confidence and reduce concerns similarly includes evidence-based interactive education. For example, after an interactive intervention with reflective learning, GPs reported expecting health benefits for their patients from genomics (50).

#### 3.1.4 Major needs identified beyond education alone

While effective education was highlighted as a major need, lack of time for the counselling and discussion of genetics required (19, 20, 30) and lack of financial support (24) and resources in terms of access to genetics advice and services (51, 52) were also reported as barriers to delivering genomics in primary care (TDF: Environmental context and resources). Cost constraints and the availability of human resources were barriers to the expansion and sustainability of education and services (TDF: Environmental context and resources). Enablers included funding for time for GPs to spend on genetic services (30) and establishing links to local genetics services support (20, 26).

Appropriate referral of patients to genetic services remains a key role for PCPs. Barriers to appropriate referral were attributed to a lack of awareness of indications for referral (32, 34, 53) and uncertainty about their role (47, 48), with many requesting referral guidelines and education (14, 21, 36) (TDF: Knowledge; TDF: Environmental context and resources). In one study (44), education increased intention to refer but not appropriate genetics referrals. The impact of education on appropriate referral was otherwise not reported. Enablers included having clear referral pathways in genetics (21) and accessible resources (54), including risk assessment tools. For example, the CRC Risk Triage tool was found to significantly increase confidence in referral (45). Additional enablers include primary-care genetics-led education, as GPs who attended a genetic counsellor-led practice-based seminar, which included referral access details and guidelines, increased appropriate referral of patients at high genetic risk of developing cancer (55). No significant changes were found for non-cancer referrals. The delivery of knowledge as a cycle rather than a one-off event was recommended for impact (55, 56), and other potential solutions included providing access to a community of practice or multidisciplinary team model (36) and being part of a genomics team (51).

## 4 Discussion

This review synthesises current attitudes to and educational interventions for genomics in primary care, identifying barriers and enablers associated with building capacity for delivery. Most studies in this review focused on aspects related to TDF domains of knowledge, environmental context and resources, professional role and identity, beliefs about capabilities, and beliefs about consequences, reflecting priority areas for PCPs. We identified four key implications (themes) associated with barriers and enablers that include knowledge as a major barrier and enabler to genomics in primary care, education as an approach to building capacity, uncertainty about the role of GPs in genomics, and major needs identified beyond education alone. These have implications for resource development, including investing in evidence-based education, alternate modes of delivery, and creating pathways and links to genetic services support. Considering the many barriers and enablers identified, it is imperative to continue to further explore and develop strategies that effectively build capacity.

When we compare our findings to a previous systematic review of genetics in primary care from almost a decade ago (6), many similar themes arise, even though only one article (34) overlaps with this 2015 review. Barriers most frequently mentioned in the systematic review by primary-care providers included a lack of knowledge (most frequently cited) about genetics and genetic risk assessment, concern for patient anxiety, a lack of access to genetics, and a lack of time—which are much the same as the barriers identified here. It is striking how similar their findings and concerns were, including the risk of genetic discrimination and harm and the lack of referral guidelines for access to genetics services, even for articles written before the genomics era.

The similarities, despite the passing of 10 years of additional genomic education, programs, and efforts to improve uptake into primary care internationally, reflect that there are systemic issues beyond education alone and that maybe a new approach is required. Moreover, as these efforts to integrate genomics and promise ‘precision’ or ‘personalized’ medicine continue, there is evidence of ambivalence and scepticism in the primary care sector, as these promises often fail to deliver (57) and may actually worsen existing inequities. Instead of more promises and programs to deliver this, there is increasing evidence that undertaking co-design (58) in partnerships with consumers and PCPs is needed (59), and incorporating more genetic skills experiences into primary care training may be required (60). Moreover, addressing the many social challenges, such as ethical and legal aspects of genomics, public acceptance, and costs, is required to enable systemic change and improve uptake in the sector (61).

In the coming decade, it could be argued that the role of the PCP is even greater in genomics, with access to more testing and guidelines and the consequent growing importance of identifying patients who would benefit from further genetic evaluation (62). Moreover, there is evidence of the important role PCPs have, as consumers value their involvement in the genomic testing process (63) and trust their PCPs to provide genomic advice and information (64). However, our findings report PCP’s ongoing concern about their role in genomics and a lack of access to genetics expertise and services, possibly reflecting the lack of effective interventions to address these longstanding problems and the rapidly changing new applications of genomics.

Genomic ‘mainstreaming’ has been promoted in many areas as a potential solution by integrating genomics into non-genetics healthcare practices such as in nursing, subspecialist physicians (10), and primary care. In the mainstreaming literature, including cancer mainstreaming literature, similar barriers have also been identified in secondary and tertiary care sectors to the uptake of genomics, including low genomic literacy and knowledge (65) and the lack of strong evidence on the type of educational interventions that lead to effective behaviour change. Some additional non-education interventions identified to impact mainstreaming include family history and referral tools, as well as embedding of genetics staff (e.g., genetic counsellors) into non-genetics areas. This can be challenging in primary care, where referral criteria and tools are very location-specific, and the low number of genetics services compared to PCPs makes it very difficult to scale up an embedded clinical service.

While genomics is a rapidly growing field with many new applications in primary care, it is helpful to compare our findings to the broader general literature on PCP education and behaviour change. A recent systematic review of reviews on primary care practitioner behaviour change using TDF has demonstrated very similar findings, with knowledge being the most frequently identified barrier and enabler identified in the primary care literature in general (66). Poor knowledge was identified as leading to uncertainty, low confidence, and poor awareness amongst PCPs. Despite this, there is literature pointing toward little apparent change in practice behaviour, even with targeted education in genetics and genomics from our study (67). This highlights the importance of evidence-based educational interventions and blended learning approaches (68) that deal with behaviour change beyond knowledge improvement.

In addition, the time and workload required to change behaviours, combined with poor resourcing and lack of time to upskill, and follow guidelines, is a major barrier identified in the primary care literature in general (69, 70). While we have focussed on just one area—genomics—a similar theme is emerging across the field, with major implications for primary care training, practice, and the way the primary care sector can adapt and change to the evolving evidence in medicine. For example, including skills-based genomics education in the training program curriculum for physicians specialising in primary care could address many of the shortfalls in the knowledge that are so common across the sector. Moreover, the time and resourcing issue speaks to a broader problem in the sector of short consultations (69) and low renumeration for time-intensive tasks such as counselling and discussing complex interventions such as genomics with patients. This is potentially compounded for discussions with patients from a non-English speaking, rural/remote, or socioeconomically disadvantaged background, where genomics may be a low priority (71). Any new interventions to address genomics uptake in primary care must also be implemented in the context of a time- and resource-poor clinician seeking quick answers to help manage their patients and broader systemic issues such as equity and training.

In the primary care literature, important social influence enablers were patient-centred care and collaboration with specialists, which is similar to the idea of a ‘community of practice’ raised as an environmental context influence enabler in our review. These are groups with a shared concern, set of problems, and regular interactions to address this and have been shown to improve primary care outcomes (72). Other models of interdisciplinary care, such as genomic multidisciplinary teams where clinical geneticists and genetic counsellors partner with non-genetics professionals to handle genomic cases and facilitate mainstreaming, are also worth considering (73, 74). Both approaches are worth exploring to enable PCPs to facilitate genomics in primary care, with genetics support and patient-centred collaborations to improve outcomes.

A strength of this review is the use of a comprehensive search strategy across multiple databases to understand the education needs, gaps, and enablers for building capacity in genomics within primary care. However, in our initial search, studies were excluded if they did not discuss genomics in the context of education, As a result, we may not have adequately captured literature related to relevant issues explored in genomics.

Moreover, we limited our definition of primary care practitioners to closely align with the Australian context of general practitioners, which excluded some physicians that are considered part of primary care in other jurisdictions, such as internal medicine and paediatric physicians. These specialties are already well represented in other research on genomic mainstreaming needs, mainly in the tertiary sector (10), but we acknowledge that this limits some of the findings to the family physician context.

Only a minority of studies in the genomics education and primary care literature utilised any theory-based frameworks or implementation science, such as using the TDF. Such use of theories is helpful for consistency across the literature and in devising interventions to address the barriers, which seem to be common across the primary care literature as a whole rather than specific to genetics itself. A potential area of future study would be the types of interventions best suited to implementing genomics beyond conventional education alone; for example, audit and feedback have been used to enable prescribing behaviour change (75), and the emergence of artificial intelligence/virtual reality-based learning tools are worth exploring further.

Although PCPs report optimism about the benefits of genomics and interest in genomics education, longstanding entrenched barriers to the delivery of genomics in primary care remain. Ensuring that education strategies are multifaceted and evidence-based and include interactive components to change behaviour will help address these barriers. Clarifying the role of the GP in training curricula, resourcing for genomics, providing clearer referral pathways, and establishing links to local genetics services support could be expected to further help the delivery of genomics in primary care. Moreover, the emergence of AI tools in practice management software and education, as well as the role of the genetic counsellor in primary care, are worth exploring in future studies. Such strategies call for close collaboration between primary care and tertiary-based genetics services to facilitate education, and even a community of practice for GPs in genomics, as a key step toward building capacity.

## Data Availability

The original contributions presented in the study are included in the article/Supplementary material, further inquiries can be directed to the corresponding author.

## On behalf of the PRECISE project team

Kristi J. Jones, Sydney Children’s Hospitals Network; Robyn Jamieson, Sydney Children’s Hospitals Network, Children’s Medical Research Institute; Nicole Rankin, The University of Melbourne; Stephen Barnett, General Practice, The University of Wollongong, Medcast; Kirsten Boggs, Murdoch Children’s Research Institute; Edwina Middleton, Centre for Genetics Education, HETI, NSW Health; Emma Bonser, Genetic Alliance Australia; Jan Mumford, Consumer Advocate; Anthony Brown, Health Consumers NSW; Caitlin Forwood, Clinical Genetics, Northern Sydney Local Health District; Alexandra Williams, HealthPathways, Nepean Blue Mountains Primary Health Network; Fi Lam, General Practice; Nick Rosser, Nepean Blue Mountains Health Pathways; Kate Baker-Marges, General Practitioner, Nepean Blue Mountains HealthPathways; Mehrnoush Bonakdar Tehrani, Postdoctoral Fellow, PRECISE, University of Sydney; Janette Hayward, Genetic Counsellor, PRECISE, Sydney Children’s Hospitals Network.

## References

[1] O'Shea R Ma AS Jamieson RV Rankin NM. Precision medicine in Australia: now is the time to get it right. Med J Aust. (2022) 217:559–63. doi: 10.5694/mja2.51777, PMID: 36436133 PMC10100177

[2] Hayward J Bishop M Rafi I Davison V. Genomics in routine clinical care: what does this mean for primary care? Br J Gen Pract. (2017) 67:58–9. doi: 10.3399/bjgp17X688945, PMID: 28126856 PMC5308090

[3] Parliment A. The new frontier - delivering better health for all Australians: Parliment of Australia; (2021) Available online at:https://www.aph.gov.au/Parliamentary_Business/Committees/House/Health_Aged_Care_and_Sport/Newdrugs/Report.

[4] WHO. Primary care: World Health Organisation; (2023). Available online at:https://www.who.int/health-topics/primary-health-care#tab=tab_1.

[5] Cusack MB Hickerton C Nisselle A McClaren B Terrill B Gaff C . General practitioners' views on genomics, practice and education: a qualitative interview study. Aust J Gen Pract. (2021) 50:747–52. doi: 10.31128/AJGP-05-20-5448, PMID: 34590089

[6] Mikat-Stevens NA Larson IA Tarini BA. Primary-care providers’ perceived barriers to integration of genetics services: a systematic review of the literature. Genet Med. (2015) 17:169–76. doi: 10.1038/gim.2014.101 25210938

[7] Larson EA Wilke RA. Integration of genomics in primary care. Am J Med. (2015) 128:1251. doi: 10.1016/j.amjmed.2015.05.011 26031886

[8] Hayward J Evans W Miller E Rafi I. Embedding genomics across the NHS: a primary care perspective. Future Healthcare J. (2023) 10:263–9. doi: 10.7861/fhj.2023-0116, PMID: 38162198 PMC10753202

[9] Tricco AC Lillie E Zarin W O'Brien KK Colquhoun H Levac D . PRISMA extension for scoping reviews (PRISMA-ScR): checklist and explanation. Ann Intern Med. (2018) 169:467–73. doi: 10.7326/M18-0850, PMID: 30178033

[10] White S Jacobs C Phillips J. Mainstreaming genetics and genomics: a systematic review of the barriers and facilitators for nurses and physicians in secondary and tertiary care. Genet Med. (2020) 22:1149–55. doi: 10.1038/s41436-020-0785-6 32313152

[11] Atkins L Francis J Islam R O’Connor D Patey A Ivers N . A guide to using the theoretical domains framework of behaviour change to investigate implementation problems. Implement Sci. (2017) 12:1–18. doi: 10.1186/s13012-017-0605-9 28637486 PMC5480145

[12] Horowitz CR Orlando LA Slavotinek AM Peterson J Angelo F Biesecker B . The genomic medicine integrative research framework: a conceptual framework for conducting genomic medicine research. Am J Hum Genet. (2019) 104:1088–96. doi: 10.1016/j.ajhg.2019.04.006, PMID: 31104772 PMC6556906

[13] Hauser D Obeng AO Fei K Ramos MA Horowitz CR. Views of primary care providers on testing patients for genetic risks for common chronic diseases. Health Aff (Millwood). (2018) 37:793–800. doi: 10.1377/hlthaff.2017.1548, PMID: 29733703 PMC6503526

[14] Carroll JC Allanson J Morrison S Miller FA Wilson BJ Permaul JA . Informing integration of genomic medicine into primary care: An assessment of current practice, attitudes, and desired resources. Front Genet. (2019) 10:1189. doi: 10.3389/fgene.2019.01189, PMID: 31824576 PMC6882282

[15] Bernhardt BA Zayac C Gordon ES Wawak L Pyeritz RE Gollust SE. Incorporating direct-to-consumer genomic information into patient care: attitudes and experiences of primary care physicians. Per Med. (2012) 9:683–92. doi: 10.2217/pme.12.80, PMID: 23795206 PMC3684987

[16] Leitsalu L Hercher L Metspalu A. Giving and withholding of information following genomic screening: challenges identified in a study of primary care physicians in Estonia. J Genet Couns. (2012) 21:591–604. doi: 10.1007/s10897-011-9424-3, PMID: 22160497

[17] Lemke AA Amendola LM Kuchta K Dunnenberger HM Thompson J Johnson C . Primary care physician experiences with integrated population-scale genetic testing: a mixed-methods assessment. J Pers Med. (2020) 10:165. doi: 10.3390/jpm10040165, PMID: 33066060 PMC7720124

[18] Morberg Jamterud S Snoek A van Langen IM Verkerk M Zeiler K. Qualitative study of GPs' views and experiences of population-based preconception expanded carrier screening in the Netherlands: bioethical perspectives. BMJ Open. (2021) 11:e056869. doi: 10.1136/bmjopen-2021-056869, PMID: 34887284 PMC8663082

[19] Yu MWC Fung JLF Ng APP Li Z Lan W Chung CCY . Preparing genomic revolution: attitudes, clinical practice, and training needs in delivering genetic counseling in primary care in Hong Kong and Shenzhen, China. Mol Genet Genomic Med. (2021) 9:e1702. doi: 10.1002/mgg3.1702, PMID: 34002545 PMC8372068

[20] Best S Long JC Fehlberg Z Theodorou T Hatem S Archibald A . The more you do it, the easier it gets: using behaviour change theory to support health care professionals offering reproductive genetic carrier screening. Eur J Hum Genet. (2023) 31:430–44. doi: 10.1038/s41431-022-01224-5, PMID: 36424524 PMC9686264

[21] Harding B Webber C Ruhland L Dalgarno N Armour C Birtwhistle R . Bridging the gap in genetics: a progressive model for primary to specialist care. BMC Med Educ. (2019) 19:195. doi: 10.1186/s12909-019-1622-y, PMID: 31185964 PMC6558677

[22] Marathe JA Woodroffe J Ogden K Hughes C. General Practitioners' knowledge and use of genetic counselling in managing patients with genetic cardiac disease in non-specialised settings. J Community Genet. (2015) 6:375–82. doi: 10.1007/s12687-015-0229-1, PMID: 25963807 PMC4567985

[23] Fok RW Ong CSB Lie D Ishak D Fung SM Tang WE . How practice setting affects family physicians' views on genetic screening: a qualitative study. BMC Fam Pract. (2021) 22:141. doi: 10.1186/s12875-021-01492-y, PMID: 34210270 PMC8247620

[24] Mitchell S Jaccard E Schmitz FM von Kanel E Collombet P Cornuz J . Investigating acceptability of a training programme in precision medicine for frontline healthcare professionals: a mixed methods study. BMC Med Educ. (2022) 22:556. doi: 10.1186/s12909-022-03613-2, PMID: 35850770 PMC9294840

[25] Melo DG de Paula PK de Araujo RS da Silva de Avo LR Germano CM Demarzo MM. Genetics in primary health care and the National Policy on Comprehensive Care for People with rare diseases in Brazil: opportunities and challenges for professional education. J Community Genet. (2015) 6:231–40. doi: 10.1007/s12687-015-0224-6, PMID: 25893505 PMC4524835

[26] Ayoub A Lapointe J Nabi H Pashayan N. Risk-stratified breast Cancer screening incorporating a polygenic risk score: a survey of UK general Practitioners' knowledge and attitudes. Genes (Basel). (2023) 14:732. doi: 10.3390/genes14030732, PMID: 36981003 PMC10048009

[27] Smit AK Newson AJ Keogh L Best M Dunlop K Vuong K . GP attitudes to and expectations for providing personal genomic risk information to the public: a qualitative study. BJGP Open. (2019) 3. doi: 10.3399/bjgpopen18X101633 PMC6480852 31049413

[28] Wilson BJ Islam R Francis JJ Grimshaw JM Permaul JA Allanson JE . Supporting genetics in primary care: investigating how theory can inform professional education. Eur J Hum Genet. (2016) 24:1541–6. doi: 10.1038/ejhg.2016.68, PMID: 27329737 PMC5110065

[29] Baroncini A Calabrese O Colotto M Pelo E Torricelli F Boccia S. Knowledge and attitude of general pratictioners towards direct-to-consumer genomic tests: a survey conducted in Italy. Epidemiol Biostat Public Health. (2015) 12. doi: 10.2427/11613

[30] Best S Long JC Fehlberg Z Archibald AD Braithwaite J. Supporting healthcare professionals to offer reproductive genetic carrier screening: a behaviour change theory approach. Aust J Prim Health. (2023) 29:480–9. doi: 10.1071/PY23022, PMID: 37156638

[31] Van Wyk C Wessels TM Kromberg JG Krause A. Knowledge regarding basic concepts of hereditary cancers, and the available genetic counselling and testing services: a survey of general practitioners in Johannesburg, South Africa. S Afr Med J. (2016) 106:268–71. doi: 10.7196/SAMJ.2016.v106i3.10162, PMID: 26915940

[32] Nair N Bellcross C Haddad L Martin M Matthews R Gabram-Mendola S . Georgia primary care Providers' knowledge of hereditary breast and ovarian Cancer syndrome. J Cancer Educ. (2017) 32:119–24. doi: 10.1007/s13187-015-0950-9, PMID: 26637472

[33] Houwink EJ Henneman L Westerneng M van Luijk SJ Cornel MC Dinant JG . Prioritization of future genetics education for general practitioners: a Delphi study. Genet Med. (2012) 14:323–9. doi: 10.1038/gim.2011.15, PMID: 22241093 PMC3905703

[34] Houwink EJ van Luijk SJ Henneman L van der Vleuten C Jan Dinant G Cornel MC. Genetic educational needs and the role of genetics in primary care: a focus group study with multiple perspectives. BMC Fam Pract. (2011) 12:1–9. doi: 10.1186/1471-2296-12-5 21329524 PMC3053218

[35] Skinner SJ Clay AT McCarron MCE Liskowich S. Interpretation and management of genetic test results by Canadian family physicians: a multiple choice survey of performance. J Community Genet. (2021) 12:479–84. doi: 10.1007/s12687-021-00511-w, PMID: 33619689 PMC8241956

[36] Terrill BN Pearce A Chau A Young M-A. Navigating genomic testing: evaluation of an e-learning module with general practitioners. Focus Health Profes Educ. (2024) 25:37–50. doi: 10.11157/fohpe.v25i1.630

[37] Vieira TA Giugliani C da Silva LP Faccini LS Loguercio Leite JC Artigalas OA . Inclusion of medical genetics in primary health care: report of a pilot project in Brazil. J Community Genet. (2013) 4:137–45. doi: 10.1007/s12687-012-0110-4, PMID: 22829114 PMC3537966

[38] Carroll JC Wilson BJ Allanson J Grimshaw J Blaine SM Meschino WS . GenetiKit: a randomized controlled trial to enhance delivery of genetics services by family physicians. Fam Pract. (2011) 28:615–23. doi: 10.1093/fampra/cmr040, PMID: 21746696

[39] Telner D Carroll JC Regehr G Tabak D Semotiuk K Freeman R. Teaching primary care genetics: a randomized controlled trial comparison. Fam Med. (2017) 49:443–50. PMID: 28633170

[40] Houwink EJ van Teeffelen SR Muijtjens AM Henneman L Jacobi F van Luijk SJ . Sustained effects of online genetics education: a randomized controlled trial on oncogenetics. Eur J Hum Genet. (2014) 22:310–6. doi: 10.1038/ejhg.2013.163, PMID: 23942200 PMC3925286

[41] Jackson L O'Connor A Paneque M Curtisova V Lunt PW Pourova RK . The gen-equip project: evaluation and impact of genetics e-learning resources for primary care in six European languages. Genet Med. (2019) 21:718–26. doi: 10.1038/s41436-018-0132-3, PMID: 30050101 PMC6752302

[42] Calabro GE Tognetto A Mazzaccara A Barbina D Carbone P Guerrera D . Capacity building of health professionals on genetics and genomics practice: evaluation of the effectiveness of a distance learning training course for Italian physicians. Front Genet. (2021) 12:626685. doi: 10.3389/fgene.2021.626685, PMID: 33790945 PMC8005606

[43] Bell RA McDermott H Fancher TL Green MJ Day FC Wilkes MS. Impact of a randomized controlled educational trial to improve physician practice behaviors around screening for inherited breast cancer. J Gen Intern Med. (2015) 30:334–41. doi: 10.1007/s11606-014-3113-5, PMID: 25451990 PMC4351290

[44] Houwink EJ Muijtjens AM van Teeffelen SR Henneman L Rethans JJ Jacobi F . Effect of comprehensive oncogenetics training interventions for general practitioners, evaluated at multiple performance levels. PLoS One. (2015) 10:e0122648. doi: 10.1371/journal.pone.0122648, PMID: 25837634 PMC4383330

[45] Carroll JC Blaine S Permaul J Dicks E Warner E Esplen MJ . Efficacy of an educational intervention on family physicians' risk assessment and management of colorectal cancer. J Community Genet. (2014) 5:303–11. doi: 10.1007/s12687-014-0185-1, PMID: 24715212 PMC4159475

[46] Houwink EJ Muijtjens AM van Teeffelen SR Henneman L Rethans JJ van der Jagt LE . Effectiveness of oncogenetics training on general practitioners' consultation skills: a randomized controlled trial. Genet Med. (2014) 16:45–52. doi: 10.1038/gim.2013.69, PMID: 23722870 PMC3914027

[47] van Vliet ME Kerkhoffs JH Harteveld CL Houwink EJF. Hemoglobinopathy screening in primary care in the Netherlands: exploring the problems and needs of patients and general practitioners. Eur J Hum Genet. (2023) 31:417–23. doi: 10.1038/s41431-022-01156-0, PMID: 35945245 PMC10133269

[48] Tan YY Spurdle AB Obermair A. Knowledge, attitudes and referral patterns of lynch syndrome: a survey of clinicians in Australia. J Pers Med. (2014) 4:218–44. doi: 10.3390/jpm4020218, PMID: 25563224 PMC4263974

[49] Vassy JL Kerman BJ Harris EJ Lemke AA Clayman ML Antwi AA . Perceived benefits and barriers to implementing precision preventive care: results of a national physician survey. Eur J Hum Genet. (2023) 31:1309–16. doi: 10.1038/s41431-023-01318-8, PMID: 36807341 PMC10620193

[50] Carroll JC Makuwaza T Manca DP Sopcak N Permaul JA O’Brien MA . Primary care providers’ experiences with and perceptions of personalized genomic medicine. Can Fam Physician. (2016) 62:e626–35. PMID: 27737998 PMC5063789

[51] Carroll JC Morrison S Miller FA Wilson BJ Permaul JA Allanson J. Anticipating the primary care role in genomic medicine: expectations of genetics health professionals. J Community Genet. (2021) 12:559–68. doi: 10.1007/s12687-021-00544-1, PMID: 34379295 PMC8554873

[52] Rafi I Crinson I Dawes M Rafi D Pirmohamed M Walter FM. The implementation of pharmacogenomics into UK general practice: a qualitative study exploring barriers, challenges and opportunities. J Community Genet. (2020) 11:269–77. doi: 10.1007/s12687-020-00468-2, PMID: 32468238 PMC7295877

[53] Presutti RJ Pujalte GGA Woodruff A Agarwal A Robinson CN Reese RL . Do physicians know when to refer patients for genetic testing? J Genet Couns. (2023) 33:786–792. doi: 10.1002/jgc4.1787 37688297

[54] Evans WRH Tranter J Rafi I Hayward J Qureshi N. How genomic information is accessed in clinical practice: an electronic survey of UK general practitioners. J Community Genet. (2020) 11:377–86. doi: 10.1007/s12687-020-00457-5, PMID: 32125658 PMC7295869

[55] Westwood G Pickering R Latter S Little P Gerard K Lucassen A . A primary care specialist genetics service: a cluster-randomised factorial trial. Br J Gen Pract. (2012) 62:e191–7. doi: 10.3399/bjgp12X630089, PMID: 22429436 PMC3289825

[56] Barreiro CZ Bidondo MP Garrido JA Deurloo J Acevedo E Luna A . CHACO outreach project: the development of a primary health care-based medical genetic service in an Argentinean province. J Community Genet. (2013) 4:321–34. doi: 10.1007/s12687-013-0157-x, PMID: 23904211 PMC3739855

[57] Pot M Spalletta O Green S. Precision medicine in primary care: how GPs envision “old” and “new” forms of personalization. Soc Sci Med. (2024) 358:117259. doi: 10.1016/j.socscimed.2024.117259, PMID: 39181083

[58] Frost A Kelly A Bishop M Bogue D Copson E Gompertz L . Genotes–a ‘just-in-time’genomics education resource co-designed with clinicians. BMC Med Educ. (2024) 24:1378. doi: 10.1186/s12909-024-06059-w, PMID: 39593035 PMC11600734

[59] Evans W Meslin EM Kai J Qureshi N. Precision medicine—are we there yet? A narrative review of precision medicine’s applicability in primary care. J Personal Med. (2024) 14:418. doi: 10.3390/jpm14040418, PMID: 38673045 PMC11051552

[60] Falah N Umer A Warnick E Vallejo M Lefeber T. Genetics education in primary care residency training: satisfaction and current barriers. BMC Primary Care. (2022) 23:156. doi: 10.1186/s12875-022-01765-0, PMID: 35718772 PMC9208192

[61] Mai C-W Sridhar SB Karattuthodi MS Ganesan PM Shareef J Lee EL . Scoping review of enablers and challenges of implementing pharmacogenomics testing in the primary care settings. BMJ Open. (2024) 14:e087064. doi: 10.1136/bmjopen-2024-087064, PMID: 39500605 PMC11552560

[62] Hull LE Gold NB Armstrong KA. Revisiting the roles of primary care clinicians in genetic medicine. JAMA. (2020) 324:1607–8. doi: 10.1001/jama.2020.18745, PMID: 32970138

[63] Puryear L Downs N Nevedal A Lewis ET Ormond KE Bregendahl M . Patient and provider perspectives on the development of personalized medicine: a mixed-methods approach. J Community Genet. (2018) 9:283–91. doi: 10.1007/s12687-017-0349-x 29280052 PMC6002302

[64] Miller FA Carroll JC Wilson BJ Bytautas JP Allanson J Cappelli M . The primary care physician role in cancer genetics: a qualitative study of patient experience. Fam Pract. (2010) 27:563–9. doi: 10.1093/fampra/cmq035, PMID: 20534792

[65] Morrow A Chan P Tucker KM Taylor N. The design, implementation, and effectiveness of intervention strategies aimed at improving genetic referral practices: a systematic review of the literature. Genet Med. (2021) 23:2239–49. doi: 10.1038/s41436-021-01272-0, PMID: 34426665 PMC8629749

[66] Mather M Pettigrew LM Navaratnam S. Barriers and facilitators to clinical behaviour change by primary care practitioners: a theory-informed systematic review of reviews using the theoretical domains framework and behaviour change wheel. Syst Rev. (2022) 11:180. doi: 10.1186/s13643-022-02030-2, PMID: 36042457 PMC9429279

[67] Paneque M Turchetti D Jackson L Lunt P Houwink E Skirton H. A systematic review of interventions to provide genetics education for primary care. BMC Fam Pract. (2016) 17:89. doi: 10.1186/s12875-016-0483-2, PMID: 27445117 PMC4957387

[68] Lyu X Li S. Professional medical education approaches: mobilizing evidence for clinicians. Front Med. (2023) 10:1071545. doi: 10.3389/fmed.2023.1071545, PMID: 37575990 PMC10419302

[69] Fiscella K Epstein RM. So much to do, so little time: care for the socially disadvantaged and the 15-minute visit. Arch Intern Med. (2008) 168:1843–52. doi: 10.1001/archinte.168.17.1843, PMID: 18809810 PMC2606692

[70] Holmér S Nedlund A-C Thomas K Krevers B. How health care professionals handle limited resources in primary care – an interview study. BMC Health Serv Res. (2023) 23:6. doi: 10.1186/s12913-022-08996-y, PMID: 36597086 PMC9808951

[71] Best S Vidic N An K Collins F White SM. A systematic review of geographical inequities for accessing clinical genomic and genetic services for non-cancer related rare disease. Eur J Hum Genet. (2022) 30:645–52. doi: 10.1038/s41431-021-01022-5, PMID: 35046503 PMC9177836

[72] Noar AP Jeffery HE Subbiah Ponniah H Jaffer U. The aims and effectiveness of communities of practice in healthcare: a systematic review. PLoS One. (2023) 18:e0292343. doi: 10.1371/journal.pone.0292343, PMID: 37815986 PMC10564133

[73] Ma A O’Shea R Wedd L Wong C Jamieson RV Rankin N. What is the power of a genomic multidisciplinary team approach? A systematic review of implementation and sustainability. Eur J Hum Genet. (2024) 32:381–91. doi: 10.1038/s41431-024-01555-5, PMID: 38378794 PMC10999446

[74] Ma A Newing TP O'Shea R Gokoolparsadh A Murdoch E Hayward J . Genomic multidisciplinary teams: a model for navigating genetic mainstreaming and precision medicine. J Paediatr Child Health. (2024) 60:118–24. doi: 10.1111/jpc.16547, PMID: 38605555

[75] Alderson SL Bald A Carder P Farrin A Foy R. Establishing a primary care audit and feedback implementation laboratory: a consensus study. Implement. Sci. Commun. (2021) 2:3. doi: 10.1186/s43058-020-00103-8, PMID: 33413700 PMC7792204

[76] Carroll JC Grad R Allanson JE Pluye P Permaul JA Pimlott N . The gene messenger impact project: An innovative genetics continuing education strategy for primary care providers. J Contin Educ Heal Prof. (2016) 36:178–85. doi: 10.1097/CEH.0000000000000079 27583994

[77] Hansen CA Reiter AW Wildin RS. Growth in perceived clinical genetics competency among primary care providers participating in genomic population health screening. J Community Genet. (2024) 15:33–7. doi: 10.1007/s12687-023-00675-7, PMID: 37792155 PMC10857985

[78] Haga SB Burke W Ginsburg GS Mills R Agans R. Primary care physicians\u0027 knowledge of and experience with pharmacogenetic testing. Clin Genet. (2012) 82:388–94. doi: 10.1111/j.1399-0004.2012.01908.x 22698141 PMC3440554

[79] Sebastian A Carroll JC Vanstone M Clausen M Kodida R Reble E. Challenges and practical solutions for managing secondary genomic findings in primary care. Eur J Med Genet. (2022) 65:104384. doi: 10.1016/j.ejmg.2021.104384 34768014

[80] Dormandy E Reid E Tsianakas V O\u0027Neil B Gill E Marteau TM. Offering antenatal sickle cell and thalassaemia screening in primary care: A pre-post evaluation of a brief type of communication skills training. Patient Educ Couns. (2012) 89:129–33. doi: 10.1016/j.pec.2012.05.004 22742984

[81] Brown-Johnson CG Safaeinili N Baratta J Palaniappan L Mahoney M Rosas LG . Implementation outcomes of Humanwide: integrated precision health in team-based family practice primary care. BMC Fam Pract. (2021) 22:28. doi: 10.1186/s12875-021-01373-4 33530939 PMC7856755

[82] Jackson L Goldsmith L Skirton H. Guidance for patients considering direct-to-consumer genetic testing and health professionals involved in their care: development of a practical decision tool. Fam Pract. (2014) 31:341–8. doi: 10.1093/fampra/cmt087 24473677

