# Supplementary material for: Building capacity for genomics in primary care: a scoping review of practitioner attitudes, education needs, and enablers
Source: Front Med (Lausanne). 2025 Apr 30;12:1577958. doi: 10.3389/fmed.2025.1577958 (PMC12076481; doi:10.3389/fmed.2025.1577958)
Supplement: Supplementary file 1 [file Table_1.DOCX]

**Supplementary Files**

**Table S1** (Supplementary Table 1). Example search string (replicated across each database)

**Table S2** (Supplementary Table 2): Demographic and study characteristics of all included studies

**Table S3** (Supplementary Table 3). Frequency of TDF domains reported in the scoping review

**Table S4** (Supplementary Table 4). TDF barriers, enablers and needs coded to the Genomic Medicine Integrative Research Framework (GMIR): Attitudes and views

**Table S5 (**Supplementary Table 5): TDF barriers, enablers and needs coded to the Genomic Medicine Integrative Research Framework (GMIR): Education and resources

**Table S1:** CINHAL Search String

(MH Genomics+)  
Genom*  
(MH Genetics+)  
Genetic*  
(MH DNA)  
DNA*  
(MH "Genetic Testing") OR (MH "Genetic Carrier Screening") OR (MH "Genetic Counselling")  
Polygenic*  
(MH "Precision Medicine+")   
"Genomic Scores"  
S1 OR S2 OR S3 OR S4 OR S5 OR S6 OR S7 OR S8 OR S9 OR S10  
(MH "Primary Health Care")  
"Primary Care*"  
"General Practice" OR (MH "General Practice") OR (MH "Physicians, Family") OR (MH "Family Practice")  
"General Prac*"  
(MH "Nurse Practitioners") OR (MH "Primary Care Nursing") OR "Primary Care Nurses"  
S12 OR S13 OR S14 OR S15 OR S16  
(MH Education+)  
Education*  
Training  
"Professional Development"  
Resources  
S18 OR S19 OR S20 OR S21 OR S22  
S11 AND S17 AND S23

"Limit 24 to yr="2011 -Current""

**Table S2:** Demographic and study characteristics of all included studies

| Study Characteristics | Frequency (N=52, %) |
| --- | --- |
| *Year of publication* | |
| 2011 - 2014 | 16 (31) |
| 2015 - 2018 | 11 (21) |
| 2019 - 2023 | 25 (48) |
| *Geographical area of study* | |
| Australia | 7 (14) |
| Asia | 2 (4) |
| Africa | 1 (2) |
| South America | 3 (6) |
| UK | 7 (14) |
| US | 10 (19) |
| Canada | 11 (21) |
| Europe | 11 (21) |
| *Key methodologies* | |
| Interviews | 8 (15) |
| Surveys | 23 (44) |
| Focus groups | 4 (8) |
| Randomised Controlled Trials | 7 (14) |
| Mixed methods | 8 (15) |
| Workshops | 2 (4) |
| *Study focus* | |
| Attitudes and views | 33 (64) |
| Education intervention | 19 (37) |
| *Context* | |
| Pre-intervention | 31 (60) |
| Post-intervention | 4 (8) |
| Pre-post intervention | 17 (33) |

**Table S3:** Frequency of TDF domains reported in the scoping review

| Domain | Frequency in articles (%) | Definition | Example |
| --- | --- | --- | --- |
| Knowledge | 34 (65.4%) | An awareness of the existence of something | Practitioners’ understanding of genomics in medicine generally and on genomics specific topics |
| Environmental context and resources | 21 (40.4%) | Any circumstance of a person's situation or environment that discourages or encourages the development of skills and abilities, independence and choose between two or more alternatives | Time, resources, processes (e.g., referral pathways) |
| Skills | 20 (38.5%) | An ability or proficiency acquired through practice | Practitioners’ ability to discuss genomics with patients |
| Professional role and identity | 17 (32.7%) | A coherent set of behaviours and displayed personal qualities of an individual in a social or work setting | Practitioners perceived role within genomics |
| Belief about capabilities | 9 (17.3%) | Acceptance of the truth, reality, or validity about an ability, talent or facility that a person can put to constructive use | Practitioner confidence in counselling patients on their genomic results |
| Optimism | 6 (11.5%) | The confidence that things will happen for the best or that desired goals will be attained | Practitioner values the role genomics will play in their future practice |
| Memory, attention, and decision processes | 7 (13.5%) | The ability to retain information, focus selectively on aspects of the environment and choose between two or more alternatives | Practitioners’ decision making on when to discuss genomics |
| Intentions | 5 (9.6%) | A conscious decision to perform a behaviour or a resolve to act in a certain way | Practitioners intentional about using risk management tools as part of their consults |
| Belief about consequences | 4 (7.7%) | Acceptance of the truth, reality, or validity about outcomes of a behaviour in a given situation | Practitioners acknowledge practical acceptability of genomics training |
| Behaviour regulation | 1 (1.9%) | Anything aimed at managing or changing objectively observed or measured actions | Practitioners incorporating reproductive genetic screening with other screening visits |
| Social influences | 1 (1.9%) | Those interpersonal processes that can cause individuals to change their thoughts, feelings, or behaviours | Practitioners interacting with other genetics practitioners for peer support |
| Emotion | 1 (1.9%) | A complex reaction pattern involving experiential, behavioural, and physiological elements, by which the individual attempts to deal with a personally significant matter or event | Increased emotional connections in consults between practitioners and patients |
| Reinforcement | 1 (1.9%) | Increasing the probability of a response by arranging a dependent relationship, or contingency, between the response and a given stimulus | Practitioners follow up with patients after initial consults |

**Table S4:** TDF barriers, enablers and needs coded to the Genomic Medicine Integrative Research Framework (GMIR): Attitudes and views (Note: Columns in this table are independent and do not correspond to the adjacent columns)

| Context | Intervention | Processes (Barriers) | Processes (Enablers) | Outcomes (Needs) | Outcomes (Implications) |
| --- | --- | --- | --- | --- | --- |
| Health care systems: Country | Type: of attitudes (33 studies) | Perceived utility, experience, reaction (findings) | Perceived utility, experience, reaction (findings) |  |  |
| Australia 6; Switzerland 1; USA 6; Canada 7; UK 3; Singapore 1; Netherlands 4; HK/China 1; South Africa 1; Italy 1; Estonia 1; Brazil 1 | Interviews 10; Focus groups 5; Survey/questionnaire 19; Delphi process 1 | Knowledge: Most general practitioners report little knowledge/skill/experience with genetics/precision medicine^1-4^ - due to lack of training, and need for more education, guidelines, resources to deal with complexities of genomics^5-10^.  It was hard to identify  good sources of information/knowledge^11^.  This issue repeated in many areas of genomics e.g. low awareness of reproductive genetic carrier screening^12^, knowledge lack, and evidence concerns around pharmacogenomics utility^13^. 49% general practitioners stated they were not familiar with polygenic risk scores^13^. In Georgia^14^, 3.9% primary care practitioners’ answered correctly about hereditary breast and ovarian cancer genetic risk, and Canadian family physicians answered poorly on survey about genetics knowledge^15^. Most participants (73%) did not feel their genetics training adequately prepared them to appropriately order or use genetic tests^16^ or deal with secondary findings^17^ or hereditary cancers^18,19^. | Primary care practitioners’ identified regular continuous professional development activities^7^ including engaging with peers and promotion via professional bodies^12^, as well as education on appropriate referrals and genetic testing^9,20^. 86.9% general practitioners reported being interested in learning more about genetic cancers^19^, and may interested in further education in this field (up to 80%^21^). | There is need for multifaceted online continuous professional development modules, linked with training program resources, and access to mentoring/advice from genetics experts and appropriate referral pathways and guidelines. Delphi process in one study^9^ highlighted need for (1) recognizing features of hereditary disease, (2) indications for referral to genetics, and (3) possibilities and limits of genetic tests. Focus groups in another study^22^ highlighted need for (1) general genetics knowledge, (2) ethical/psychosocial issues with genetic testing, and (3) role of genetics services and referral indications. | Clinician implications: There is a need for prioritised supports including continuous professional development and online resources especially in precision/personalised medicine and adding this into training programs^2^. There is also need for diverse range of resources, no 'one size fits' methodology^5^. Brazilian and US^10,21^ study pointed to need for more basic curricula in genetics and inheritance, and how this links to clinical practice. Similar needs identified in one study^15^ were patterns of inheritance, risk assessment, and understanding the benefits, risks, and limitations of genetic testing needed in teaching/curricula. |
| TOPICS: Genomics/genetics in general 8; precision medicine training; genetic competencies; reproductive carrier screening 3; direct-to-consumer testing 2; personalised medicine 1; genetic screening 2, pharmacogenomics 2; chronic disease genetic testing; genetic education; secondary findings in genomics; communicating genetic risk/polygenic scores 3 ; cancer genetics 4 ; genetic counselling in primary care ; cardiac genetics 1; haemoglobinopathy | General practitioners asked about their views, roles, needs, acceptability, barriers, and supports needed; attitudes to direct-to-consumer; access to education; bioethical concerns; knowledge; and attitudes. | Memory, attention and decision processes: Health professionals often forgetting to offer genetics due to competing priorities^12^, need for clearer referral guidelines^23^, and lack of clear referral pathways^1,18,24^. | Comprehensive websites with relevant genomics information, clear referral pathways^25^, information for patients^11^, and local genetics services as an important resource^25^. | Referral processes and need for additional genomics resources and support system. |  |
|  |  | Skill: Low skill to perform genomics (reproductive carrier screening^7^); 75.4% of general practitioners unsure on assessing patient risk of hereditary cancer^19^. |  |  |  |
|  |  |  | Behaviour regulation (habits): Making reproductive carrier screening part of other screening e.g., cervical, habitual^12^. |  |  |
|  |  | Environmental context and resources (TDF): Lack of time is a major recurrent theme - extra time required to counsel patients^3,7,12^ (e.g., reproductive carrier screening). There is a lack of resources; low social awareness of genomics^12^. and financial/regional access barriers^6^. Lack of clarity around when to offer patients reproductive carrier screening^12^, access to genetics and time^23^. Concerns about extra workload of pharmacogenetics^23^. | Funding for general practitioner time for carrier screening^12^. Making education available through different modalities with access to genetic counselling support^12^ alongside referral guidelines^25^, information for patients^11^ and local genetics services as an important resource^25^. | General practitioners identified needing more time, genetic counselling, and training^14^. Better pathways, awareness of genetic services, and relevant support needed. | Health system implications: need to build awareness of genomics, address time constraints, regional equity and financial barriers including insurance issues. There is also a need to fit genomics into 'routine' practice, habitualsing it. |
|  |  |  | Social influences: Being part of a special interest group^7^; and even general practitioners as part of genomics team with genetic health professionals ^23^ ordering tests etc. |  | Need clearer referral pathways, genetic counsellors identified as favoured resource and support^11^, building skills for conversations around genomics and its benefit and point of care tools. |
|  |  | Professional role and identity: Roles not well defined yet in the profession^6^, lack of interest in doing genetic screening^12^ and some ambivalence about role in genetics^2^. Uncertain about role in screening for hemoglobinopathies^24^, 30% of general practitioners’ did not feel that their role is to identify cancer patients for genetic referral^18^. | Professional role (TDF): general practitioners’ see growing role of genomics in future^5^, and increasingly important role to take time to raise awareness of genetics^12,20^ (especially as specialists supply exceeded by demand). Some general practitioners’ report standard role in genomics such as identifying family risk and referring^8,11^. There would be more benefits if genetic health professionals and general practitioners’ worked together more^23^ and believed that they should have primary responsibility for making patients aware of a pharmacogenomic test for a drug prescribed for them^16^. | Role delineation is a gap - not well defined, no policy directions, while general practitioners see a growing role in genomics and its importance, the responsibility and role of general practitioners in this new field is still unclear. | Motivations: Ambivalence needs to be cleared; roles defined more clearly for the general practitioners. |
|  |  | Beliefs about consequences (TDF): Low relevance now but growing in future^6^. Concerns about negative impact on patients including anxiety^12^, insurance, discrimination concerns^26^, lack of guidelines, and poor motivation to do genomics. 1 in 2 primary care professionals were concerned that genetic testing would lead to insurance discrimination (in USA^4,27^ and Estonia^28^) and concerns about privacy/discrimination^29^, costs^27^ and autonomy^28^. | Primary care professionals valued giving choice, although concerns about bioethical and societal issues need to be addressed at a broader level^30^. Primary care professionals saw genetic testing as improving patient care and identifying patients needing screening^29^. 89% saw polygenic risk scores as beneficial^27^. Belief in personalized medicine was envisioned as the future goal^3^. | There is need for conversation about potential benefits and negatives of genomics in primary care, including risks and concerns about ethical issues. | Implications were around the utility of optimism to cater to belief about consequences. |
|  |  | Intentions (TDF): Religious/ethical beliefs of patients, concerns about termination^12^. | Increase in general practitioners’ intent to implement learnings in practice^31^. Acceptance of precision medicine approaches having a positive role in health and making conversations about reproductive carrier screening a priority^12^. Many family physicians have high intention of taking family history^32^. |  | Address concerns about patient harm. |
|  |  | Beliefs about capabilities (TDF): lack of confidence interpreting results and counselling skills^5^, low confidence^1,11,14^. Only around half general practitioners felt confident discussing and explaining genetic results with patients^29^ or explaining genomic risk^33^. Lack of confidence in making risk assessments in genetics^32^. 96% general practitioners felt incapable of advising patients about direct-to-consumer^34^. | General practitioners believe genomic medicine will impact healthcare in beneficial way in the future - 57% believed that genomic medicine will improve clinical outcomes within 5 years, and those more frequently ordering testing felt more confident^26^. | General practitioners need confidence in making decisions about referrals, assessing risks in patients^32^ and finding trustworthy information that they can feel confident about. | Build confidence and trust. |
|  |  |  | Optimism: Over half general practitioners expected genomics to improve health outcomes for patients^11^. 74% agreed that genetic testing is clinically useful and 70% agreed that it will improve clinical outcomes within the next 5 years^4^. In Estonia, 96% believed genetic testing improved health outcomes^28^. | There is a need for overall optimism in primary care about genomics and its role in improving healthcare. |  |

**Table S5:** TDF barriers, enablers and needs coded to the Genomic Medicine Integrative Research Framework (GMIR): Education and resources (Note: Columns in this table are independent and do not correspond to the adjacent columns)

| Context | Intervention Type | Processes (Barriers) | Processes (Enablers) | Processes (Needs) | Outcomes (Implications) |
| --- | --- | --- | --- | --- | --- |
| Health care systems: Country | Type: of education (14/19 studies) | Perceived utility, experience, reaction (findings) | Perceived utility, experience, reaction (findings) |  |  |
| Australia 1; Brazil 1; Netherlands 4; Canada 4; UK 2; USA 4; Argentina 1; Italy 1; | 1.Continous professional development module: 90 min online: informed by content framework piloted with general practitioners and general practitioners educators^31^. | Knowledge (TDF) *Barriers:* 1. Primary care providers have insufficient knowledge of genetics pre-intervention^35^ 2. No significant difference was found in knowledge retention 6 months after the lecture series program^35^. 3. No significant difference was found for knowledge for gene kit intervention^36^. | Knowledge (TDF) *Enablers to improve knowledge:* Accessible case-based online continuous professional development education module (with or without scenario) improved knowledge^31,37,38^, lecture series improved knowledge immediately post education^35^, Online module/live web based presentation or /live plus patient encounter all improved knowledge and skills^37^;  Case based oncogenetics e- continuous professional development module (accessible) sustained improved knowledge^38^; colorectal cancer triage tool increased awareness of criteria for referral^39^; Genomic Medicine Action Plan messaging tool improved knowledge for population genetic screening (87.5%)^40^; Gen-equip e-modules based on cases led to significant increase in knowledge especially colorectal cancer and pregnancy^41^: Distance learning course based on problem case-based learning improved knowledge particularly in general practitioners^42^. | Greater communication between primary care professionals and clinical geneticist for population screening^40^. | Knowledge:    Low impact of standalone lectures and resources.    Accessible case-based education improves knowledge.    Interactive, evidence-based education strategies are needed to put genomic knowledge into practice i.e., e-modules alone do not change behaviour.    Online modules provide access and help distance learning. |
|  | 2. Face-to-face lecture series^35^ delivered by genetics professionals broadly covering genomics. Topics determined by genetic health professionals and not target group. | Knowledge (TDF): *Barriers:* E-module did not have significant impact on applying knowledge i.e., for self-reported recognition of disease, referral to a specialist and knowledge of possibilities/limitations of genetic testing^38^. | Knowledge (TDF) *Enablers to put knowledge into practice:* Format of lecture + interactive scenario reported as “ideal way to learn” as the experience allowed them to put “knowledge into practice”^37^. | Further training in counselling on genetic testing for population genetic screening^40^. | Applying knowledge:    Interactive components may help change behaviour e.g., lecture plus interactive scenario is ideal. |
|  | 3. Web-based module outlining genetic concepts applied to colorectal cancer, compared to (2) live presentation of the web-based material, compared to (3) live presentation and subsequent standardized patient encounter^37^. | Knowledge (TDF) | Knowledge (TDF) *Enablers for high satisfaction:* Education format informed by evidence-based educational strategies improves knowledge and high satisfaction i.e., content informed by target group and includes case studies^31^, includes multiple methods, tools, interactive components^38^, email 'push' model with reflective learning leads to high satisfaction^8^; Gene-equip online module based on cases in six languages had high satisfaction^41^. | Regular case-based discussions for population genomic screening^40^. | Satisfaction:    Evidence-based educational strategies needed to improve knowledge and satisfaction (case studies and reflective learning have impact).    Multimodal captures different needs of general practitioners.      Providing available resources. |
|  | 4. E-continuous professional development oncogenetics module^38^: informed by Delphi, multiple educational methodologies. |  | Knowledge (TDF) *Enablers on long term impact of education:* Live continuous professional development module training had more impact on general practitioners’ being more aware of genetic problems long term^43^. |  | Long term impact:    Evidence-based education has impact, live (interactive) training has long term impact. |
|  | 5. 4-hr F2F evening oncogenetic consultation skills training; included interactive theoretical session, consumer/patient experience presentation, 2-hr role-playing 3 cases^44^. |  | Knowledge (TDF) *Enablers*: 'Distilling information into a useful and accessible “bottom line” with which to guide practice: e.g., 'Genomics fundamentals' accessible as a refresher^31^; E-mail push model 'Gene Messenger'^45^. |  | Education that is useful and accessible needs to be considered. |
|  | 6. Three oncogenetics modules (e- continuous professional development: live continuous professional development module; genetics website): an online Continuing Professional Development (G-eCPD) module, a live genetic continuous professional development module, and a “general practitioner and genetics”website^43^. | Skills | Skills (TDF) *Enablers to improve*: Key consultation skills significantly and substantially improved after F2Fskills +role play training^44^; Physicians in the intervention group had a higher referral rate than before ^36^; Assessing risk, screening method, frequency and whether screening was indicated significantly improved after colorectal cancer risk triage tool^39^. |  | Changing behaviour.    Multimodal education with online, live component and resources changes behaviour. |
|  | 7.Individual evidence-based reflective pushed e-learning  method (Gene Messengers: Email "push" model) regular email summaries of new tests/info followed by the Individual Reflective E-learning Questionnaire^45^. |  | Memory, attention and decision process: genetic counselling led primary care genetics education increased general practitioners’ appropriate referral of patients at moderate and high genetic risk of developing cancer. No significant changes for non-cancer referrals. |  | Confidence: Any type of education increases confidence. |
|  | 8. Genetikit: evidence-based summaries of genetic tests with primary care recommendations^36^. |  | Skills (TDF) *Enablers to sustain*: Online module/live training increased self-reported genetic consultation skills long term after one-year^43^; Live training 65% reported applying the newly learned (consultation) skills monthly^44^; Sustained improvement in consultation skills 3 months after face-to-face skills+-role play training^44^. |  | Sustained  Live face-to-face training increases likelihood of sustained improvement. |
|  | 9. Practice-based informal seminar detailing referral pathways, electronic referral guidelines and example case scenarios illustrating inheritance patterns, recurrence risks and ethical issues^46^. |  | Social/Professional Role and identity (TDF) *Enabler:* Genetics Health professionals presenting education to primary care enables greater appreciation of roles^35^. General practitioners’ favoured inclusion of case studies modelling  pivotal roles for general practitioners’, such as taking an accurate family history and referring appropriately  to genetics. |  | Ambivalence and ambiguity of general practitioner role in genetics.    Need to recognise the role of the general practitioner.    Genetic Health Professionals providing education can provide a link and clarity.      Being part of a special interest group may help clarify role and provide access to information. |
|  | 10. The Gen-Equip Project: nine genetics e-learning modules-based cases for primary care in six European languages^41^ (UK/Netherlands) | Beliefs about capabilities (TDF) *Barriers:* | Beliefs about capabilities (TDF) *Enablers:* Content informed by target group online module with case studies led to increase in general practitioner confidence^31^; Confidence in individual competencies was also higher for the intervention group Genetikit (summaries with recommendations)^36^; colorectal cancer risk triage tool significantly increased confidence in referral^39^; Genomic Medicine Action Plan messaging tool increased confidence in offering genetic testing and discussing results for population health screening^40^; Gen-equip (online modules based on cases led to increased confidence in talking about genetic test results and genetic testing with families and teaching^41^. |  | Confidence    Relevant information particularly based on case studies with available resources increases confidence in consultation and referral.    Best informed by target group. |
|  | 11.Genetics and Genomics practice distance learning course: focused on genetic/genomics testing, pharmacogenetics and oncogenomics.  and was developed according to andragogical training methods (Problem-based Learning and Case-based Learning)^42^. |  |  |  |  |
|  | 12.Colorectal risk triage/Management Tool (educational intervention) for triage, screening and genetic referral recommendations for patients with colorectal cancer family history^39^. | Belief about consequences (TDF)    *Barriers:* Online education does not change attitudes^31^; including module and live webinars^37^. | Belief about consequences (TDF) *Enablers:* General practitioners’ expected health benefits after Gene Messenger email" push" model^45^. |  |  |
|  | 13. Genomic Medicine Action Plan: Resource/educational intervention for population health screening: includes one hour orientation including in-person, recorded videos and written materials plus Genomic Medicine Action Plan, a patient and a provider facing messaging tool with patient results, actions, and information related to inheritance, risks and family member impact^40^. | Reinforcement | Reinforcement (TDF) *Enabler:* Receiving professional development points (Best). |  | Reinforcement – making good use of time.      Professional development points are reinforcers. |
|  | 14. Decision support tool/guidelines for those considering direct-to-consumer genetic tests encompassing varied clinical scenarios and clinical guidelines for primary care practitioners^47^. | Intentions: Only 2/7 questions in a family history questionnaire triggered primary care practitioners to intend to refer^48^.                               No significant differences in social influence, moral norms, belief in consequences. | Intentions (TDF):                               *Enablers:* Content informed by target group online module with case studies increase general practitioner intent to implement learnings in practice^31^; 80% live module evaluation attendees (v 64% online attendees) reported more frequently considered referring patients to the clinical genetics centres^43^; oncogenetics consultation skills training had high perceived applicability^44^. Colorectal cancer risk triage tool would continue to use and recommend to colleagues^39^. |  | Intentions  Relevant education informed by target group increases intent and may be recommend by primary care practitioners to others (but may not change behaviour). |
|  | 15. Family History Questionnaire: a family history tool for primary care where each question is an indicator referral to a genetics specialist^48^.  Findings showed that primary care practitioners didn’t see all questions as a trigger for genetic testing. | Memory, Attention and Decision Processes (TDF) Barrier:  Online module had no impact on referral to a specialist^38^ perception of peer support^31^;  Genetikit (summaries plus recommendations) made no significant difference in decisional difficulty^36^. | Memory, Attention and Decision Processes (TDF) *Enabler:* General practitioners who attended live training more likely to consider referring patients to clinical genetics centres^43^. |  | Referral    Decisional difficulty/ referral to specialists no changed after online module.    Intent to refer was higher after live training. |
|  |  | Environmental context and resources (TDF) *Barrier:* Expansion of face-to-face lecture program in its current format to other primary care facilities not feasible due to cost constraints and the limited availability of human resources^35^. | Environmental context and resources (TDF*) Enabler:* Convenience, time, pace of web-based module appreciated^37^; gene messenger-accessible, brief, wonderful way to stay up to date,94% wanted to continue to receive them^45^; Post education website visitors and satisfaction increased^43^; Experts (consensus workshop) identified 7 reasons why a person would present to their primary care practitioners to ask about direct-to-consumer- pathway was offered^47^; Gen-equip: -availability of resources for teaching and as a vehicle to form stronger links with primary care and genetics^41^. |  | Primary care practitioners appreciate convenience, time and pace of web-based education.    Available resources for teaching provide links to services. |
|  |  | Social influences | Social influences (TDF) *Enabler:*  Being part of a reproductive genetic carrier screening special interest group (Best). |  | Links to genetic services, comm unity of practice. |
|  |  | Emotion (TDF) *Barrier:* | Emotion (TDF) *Enabler*: Emotional connection with patients^12^. |  | Education may reduce concerns about patients. |
|  |  | Behavioural Regulation | Professional role (TDF) *Enabler:* Recognise the role of the general practitioner^31^. |  | Reflect general practitioner roles in genomics in professional bodies/curricula. |

**References**

1. Marathe JA, Woodroffe J, Ogden K, Hughes C. General Practitioners' knowledge and use of genetic counselling in managing patients with genetic cardiac disease in non-specialised settings. *J Community Genet*. Oct 2015;6(4):375-82. doi:10.1007/s12687-015-0229-1

2. Fok RW, Ong CSB, Lie D, et al. How practice setting affects family physicians' views on genetic screening: a qualitative study. *BMC Fam Pract*. Jul 1 2021;22(1):141. doi:10.1186/s12875-021-01492-y

3. Yu MWC, Fung JLF, Ng APP, et al. Preparing genomic revolution: Attitudes, clinical practice, and training needs in delivering genetic counseling in primary care in Hong Kong and Shenzhen, China. *Mol Genet Genomic Med*. Jul 2021;9(7):e1702. doi:10.1002/mgg3.1702

4. Hauser D, Obeng AO, Fei K, Ramos MA, Horowitz CR. Views Of Primary Care Providers On Testing Patients For Genetic Risks For Common Chronic Diseases. *Health Aff (Millwood)*. May 2018;37(5):793-800. doi:10.1377/hlthaff.2017.1548

5. Cusack MB, Hickerton C, Nisselle A, et al. General practitioners' views on genomics, practice and education: A qualitative interview study. *Australian Journal of General Practice*. 2021;50(10):747-752.

6. Mitchell S, Jaccard E, Schmitz FM, et al. Investigating acceptability of a training programme in precision medicine for frontline healthcare professionals: a mixed methods study. *BMC Med Educ*. Jul 19 2022;22(1):556. doi:10.1186/s12909-022-03613-2

7. Best S, Long JC, Fehlberg Z, Archibald AD, Braithwaite J. Supporting healthcare professionals to offer reproductive genetic carrier screening: a behaviour change theory approach. *Aust J Prim Health*. Oct 2023;29(5):480-489. doi:10.1071/PY23022

8. Carroll JC, Makuwaza T, Manca DP, et al. Primary care providers’ experiences with and perceptions of personalized genomic medicine. *Canadian Family Physician*. 2016;62(10):e626-e635.

9. Houwink EJ, Henneman L, Westerneng M, et al. Prioritization of future genetics education for general practitioners: a Delphi study. *Genet Med*. Mar 2012;14(3):323-9. doi:10.1038/gim.2011.15

10. Melo DG, de Paula PK, de Araujo Rodrigues S, da Silva de Avo LR, Germano CM, Demarzo MM. Genetics in primary health care and the National Policy on Comprehensive Care for People with Rare Diseases in Brazil: opportunities and challenges for professional education. *J Community Genet*. Jul 2015;6(3):231-40. doi:10.1007/s12687-015-0224-6

11. Carroll JC, Allanson J, Morrison S, et al. Informing Integration of Genomic Medicine Into Primary Care: An Assessment of Current Practice, Attitudes, and Desired Resources. *Front Genet*. 2019;10:1189. doi:10.3389/fgene.2019.01189

12. Best S, Long JC, Fehlberg Z, et al. The more you do it, the easier it gets: using behaviour change theory to support health care professionals offering reproductive genetic carrier screening. *Eur J Hum Genet*. Apr 2023;31(4):430-444. doi:10.1038/s41431-022-01224-5

13. Rafi I, Crinson I, Dawes M, Rafi D, Pirmohamed M, Walter FM. The implementation of pharmacogenomics into UK general practice: a qualitative study exploring barriers, challenges and opportunities. *J Community Genet*. Jul 2020;11(3):269-277. doi:10.1007/s12687-020-00468-2

14. Ayoub A, Lapointe J, Nabi H, Pashayan N. Risk-Stratified Breast Cancer Screening Incorporating a Polygenic Risk Score: A Survey of UK General Practitioners' Knowledge and Attitudes. *Genes (Basel)*. Mar 16 2023;14(3)doi:10.3390/genes14030732

15. Skinner SJ, Clay AT, McCarron MCE, Liskowich S. Interpretation and management of genetic test results by Canadian family physicians: a multiple choice survey of performance. *J Community Genet*. Jul 2021;12(3):479-484. doi:10.1007/s12687-021-00511-w

16. Haga SB, Burke W, Ginsburg GS, Mills R, Agans R. Primary care physicians' knowledge of and experience with pharmacogenetic testing. *Clin Genet*. Oct 2012;82(4):388-94. doi:10.1111/j.1399-0004.2012.01908.x

17. Sebastian A, Carroll JC, Vanstone M, et al. Challenges and practical solutions for managing secondary genomic findings in primary care. *Eur J Med Genet*. Jan 2022;65(1):104384. doi:10.1016/j.ejmg.2021.104384

18. Tan YY, Spurdle AB, Obermair A. Knowledge, attitudes and referral patterns of lynch syndrome: a survey of clinicians in australia. *J Pers Med*. May 12 2014;4(2):218-44. doi:10.3390/jpm4020218

19. Van Wyk C, Wessels TM, Kromberg JG, Krause A. Knowledge regarding basic concepts of hereditary cancers, and the available genetic counselling and testing services: A survey of general practitioners in Johannesburg, South Africa. *S Afr Med J*. Feb 4 2016;106(3):268-71. doi:10.7196/SAMJ.2016.v106i3.10162

20. Harding B, Webber C, Ruhland L, et al. Bridging the gap in genetics: a progressive model for primary to specialist care. *BMC Med Educ*. Jun 11 2019;19(1):195. doi:10.1186/s12909-019-1622-y

21. Nair N, Bellcross C, Haddad L, et al. Georgia Primary Care Providers' Knowledge of Hereditary Breast and Ovarian Cancer Syndrome. *J Cancer Educ*. Mar 2017;32(1):119-124. doi:10.1007/s13187-015-0950-9

22. Houwink EJ, van Luijk SJ, Henneman L, van der Vleuten C, Jan Dinant G, Cornel MC. Genetic educational needs and the role of genetics in primary care: a focus group study with multiple perspectives. *BMC family practice*. 2011;12:1-9.

23. Carroll JC, Morrison S, Miller FA, Wilson BJ, Permaul JA, Allanson J. Anticipating the primary care role in genomic medicine: expectations of genetics health professionals. *J Community Genet*. Oct 2021;12(4):559-568. doi:10.1007/s12687-021-00544-1

24. van Vliet ME, Kerkhoffs JH, Harteveld CL, Houwink EJF. Hemoglobinopathy screening in primary care in the Netherlands: exploring the problems and needs of patients and general practitioners. *Eur J Hum Genet*. Apr 2023;31(4):417-423. doi:10.1038/s41431-022-01156-0

25. Evans WRH, Tranter J, Rafi I, Hayward J, Qureshi N. How genomic information is accessed in clinical practice: an electronic survey of UK general practitioners. *J Community Genet*. Jul 2020;11(3):377-386. doi:10.1007/s12687-020-00457-5

26. Bernhardt BA, Zayac C, Gordon ES, Wawak L, Pyeritz RE, Gollust SE. Incorporating direct-to-consumer genomic information into patient care: attitudes and experiences of primary care physicians. *Per Med*. Sep 1 2012;9(7):683-692. doi:10.2217/pme.12.80

27. Vassy JL, Kerman BJ, Harris EJ, et al. Perceived benefits and barriers to implementing precision preventive care: Results of a national physician survey. *Eur J Hum Genet*. Nov 2023;31(11):1309-1316. doi:10.1038/s41431-023-01318-8

28. Leitsalu L, Hercher L, Metspalu A. Giving and withholding of information following genomic screening: challenges identified in a study of primary care physicians in Estonia. *J Genet Couns*. Aug 2012;21(4):591-604. doi:10.1007/s10897-011-9424-3

29. Lemke AA, Amendola LM, Kuchta K, et al. Primary Care Physician Experiences with Integrated Population-Scale Genetic Testing: A Mixed-Methods Assessment. *J Pers Med*. Oct 13 2020;10(4)doi:10.3390/jpm10040165

30. Morberg Jamterud S, Snoek A, van Langen IM, Verkerk M, Zeiler K. Qualitative study of GPs' views and experiences of population-based preconception expanded carrier screening in the Netherlands: bioethical perspectives. *BMJ Open*. Dec 9 2021;11(12):e056869. doi:10.1136/bmjopen-2021-056869

31. Terrill BN, Pearce A, Chau A, Young M-A. Navigating genomic testing: Evaluation of an e-learning module with general practitioners. *Focus on Health Professional Education: A Multi-Professional Journal*. 2024;25(1):37-50.

32. Wilson BJ, Islam R, Francis JJ, et al. Supporting genetics in primary care: investigating how theory can inform professional education. *Eur J Hum Genet*. Nov 2016;24(11):1541-1546. doi:10.1038/ejhg.2016.68

33. Smit AK, Newson AJ, Keogh L, et al. GP attitudes to and expectations for providing personal genomic risk information to the public: a qualitative study. *BJGP Open*. Apr 2019;3(1):bjgpopen18X101633. doi:10.3399/bjgpopen18X101633

34. Baroncini A, Calabrese O, Colotto M, Pelo E, Torricelli F, Boccia S. Knowledge and attitude of general pratictioners towards direct-to-consumer genomic tests: a survey conducted in Italy. *Epidemiology, Biostatistics, and Public Health*. 2022;12(4)doi:10.2427/11613

35. Vieira TA, Giugliani C, da Silva LP, et al. Inclusion of medical genetics in primary health care: report of a pilot project in Brazil. *J Community Genet*. Jan 2013;4(1):137-45. doi:10.1007/s12687-012-0110-4

36. Carroll JC, Wilson BJ, Allanson J, et al. GenetiKit: a randomized controlled trial to enhance delivery of genetics services by family physicians. *Fam Pract*. Dec 2011;28(6):615-23. doi:10.1093/fampra/cmr040

37. Telner D, Carroll JC, Regehr G, Tabak D, Semotiuk K, Freeman R. Teaching Primary Care Genetics: A Randomized Controlled Trial Comparison. *Family Medicine*. 2017;49(6):443-450.

38. Houwink EJ, van Teeffelen SR, Muijtjens AM, et al. Sustained effects of online genetics education: a randomized controlled trial on oncogenetics. *Eur J Hum Genet*. Mar 2014;22(3):310-6. doi:10.1038/ejhg.2013.163

39. Carroll JC, Blaine S, Permaul J, et al. Efficacy of an educational intervention on family physicians' risk assessment and management of colorectal cancer. *J Community Genet*. Oct 2014;5(4):303-11. doi:10.1007/s12687-014-0185-1

40. Hansen CA, Reiter AW, Wildin RS. Growth in perceived clinical genetics competency among primary care providers participating in genomic population health screening. *J Community Genet*. Feb 2024;15(1):33-37. doi:10.1007/s12687-023-00675-7

41. Jackson L, O'Connor A, Paneque M, et al. The Gen-Equip Project: evaluation and impact of genetics e-learning resources for primary care in six European languages. *Genet Med*. Mar 2019;21(3):718-726. doi:10.1038/s41436-018-0132-3

42. Calabro GE, Tognetto A, Mazzaccara A, et al. Capacity Building of Health Professionals on Genetics and Genomics Practice: Evaluation of the Effectiveness of a Distance Learning Training Course for Italian Physicians. *Front Genet*. 2021;12:626685. doi:10.3389/fgene.2021.626685

43. Houwink EJ, Muijtjens AM, van Teeffelen SR, et al. Effect of comprehensive oncogenetics training interventions for general practitioners, evaluated at multiple performance levels. *PLoS One*. 2015;10(4):e0122648. doi:10.1371/journal.pone.0122648

44. Houwink EJ, Muijtjens AM, van Teeffelen SR, et al. Effectiveness of oncogenetics training on general practitioners' consultation skills: a randomized controlled trial. *Genet Med*. Jan 2014;16(1):45-52. doi:10.1038/gim.2013.69

45. Carroll JC, Grad R, Allanson JE, et al. The Gene Messenger Impact Project: An Innovative Genetics Continuing Education Strategy for Primary Care Providers. *J Contin Educ Health Prof*. Summer 2016;36(3):178-85. doi:10.1097/CEH.0000000000000079

46. Westwood G, Pickering R, Latter S, et al. A primary care specialist genetics service: a cluster-randomised factorial trial. *Br J Gen Pract*. Mar 2012;62(596):e191-7. doi:10.3399/bjgp12X630089

47. Jackson L, Goldsmith L, Skirton H. Guidance for patients considering direct-to-consumer genetic testing and health professionals involved in their care: development of a practical decision tool. *Fam Pract*. Jun 2014;31(3):341-8. doi:10.1093/fampra/cmt087

48. Presutti RJ, Pujalte GGA, Woodruff A, et al. Do physicians know when to refer patients for genetic testing? *J Genet Couns*. Sep 8 2023;doi:10.1002/jgc4.1787
